# A narrative review of the economic burden of myelin oligodendrocyte glycoprotein antibody-associated disease and analogous conditions

**DOI:** 10.3389/fneur.2025.1506465

**Published:** 2025-05-30

**Authors:** Leonard Lee, Joshua Byrnes, Cathryn Hope, Hansoo Kim

**Affiliations:** ^1^Centre for Applied Health Economics, School of Medicine and Dentistry, Griffith University, Gold Coast, QLD, Australia; ^2^UCB Inc., Morrisville, NC, United States

**Keywords:** central nervous system, economic burden, disease costs, inflammatory demyelinating disease, MOGAD

## Abstract

**Background:**

Myelin oligodendrocyte glycoprotein antibody-associated disease (MOGAD) is an inflammatory demyelinating disease of the central nervous system with a serious, debilitating presentation, including residual disability after relapses.

**Objectives:**

To evaluate the economic impacts of MOGAD and analogous conditions, including direct costs, indirect costs and cost drivers.

**Design:**

Systematic literature search and narrative review.

**Data sources and methods:**

Search strings were designed to capture any study reporting health economic impacts of MOGAD or analogous autoimmune diseases of the nervous system. The costs of diagnostic tests and short- and long-term interventions were considered, and studies from both patient and institutional (public and private) perspectives were included. Searches were conducted using medical subject headings (MeSH) in PubMed in July 2023. Retrieved publications were screened initially based on title and abstract, then based on the full text. Data were extracted manually; findings are reported descriptively. All cost data were adjusted to 2024 US Dollars using the CCEMG-EPPI-Centre Cost Converter.

**Results:**

Results from 40 studies of MOGAD and analogous autoimmune neurological conditions were extracted. In the only study that included patients with MOGAD (a cost investigation from Germany in which 166 patients had neuromyelitis optica spectrum disorder and 46 had MOGAD), the mean annualised cost of illness was $94,688 (direct medical costs 43%, direct non-medical costs 34%, indirect costs 23%). Across the conditions assessed, the annual total cost of illness per patient ranged widely, from $3,690 to $507,117 (among studies that reported types of cost, the range for direct costs was $1,981–$148,388; for indirect costs, $0–$942,707). The study that included patients with MOGAD identified the need for care, number of acute attacks, unemployment and disability as independent predictors of cost. Additional cost drivers (from all the conditions) included treatments (e.g., intravenous immunoglobulin), hospitalisation, disease severity, relapses and refractory disease.

**Conclusion:**

Our search identified only one study that specifically examined costs associated with MOGAD. Results from this and studies of analogous autoimmune conditions suggest that inflammatory demyelinating diseases of the central nervous system including MOGAD are costly for the individual patient and place considerable burden on healthcare systems. Further evidence is needed for increased insight into the economic burden of MOGAD.

## Introduction

Myelin oligodendrocyte glycoprotein antibody-associated disease (MOGAD) is an inflammatory demyelinating disease of the central nervous system (CNS) (1, 2). MOGAD has some symptom overlap with other neuroinflammatory disorders such as multiple sclerosis (MS) and neuromyelitis optica spectrum disorder (NMOSD) but there are important differences between these diseases in pathophysiology, prognosis and treatment response (1–4). Accurate and timely diagnosis of MOGAD is therefore needed to ensure that patients receive optimal therapy and minimise the risk of hospitalisation due to potentially preventable disease symptoms. This requires a standardised approach with thorough consideration of the clinical presentation and history along with the findings of serological tests and intracranial and spinal imaging (3–6). Knowledge and understanding of MOGAD have increased in recent years, allowing the development of an antibody test, unique international classification of diseases code (ICD-10-CM code G37.81) and updated diagnostic criteria (1, 2, 7, 8). Although ICD coding is not universally implemented, these developments should help enable gradual improvement in the patient pathway to diagnosis and treatment, which at present can be lengthy and challenging (9). Already they have paved the way for increased attention to the management of MOGAD. Understanding the direct and indirect costs associated with the disease and its treatment will benefit healthcare systems and will be important for determining the cost-effectiveness of future candidate treatments.

Despite being a rare disease, MOGAD is expected to represent a substantial economic burden; it has a serious debilitating presentation, with residual disability after relapses. Patients may present with one or more of optic neuritis, myelitis, acute disseminated encephalomyelitis (ADEM), cerebral mono-focal or multifocal deficits, brainstem or cerebellar syndromes or cerebral cortical encephalitis (1, 2, 10). Symptomatic attacks may be attributable to inflammation of the optic nerve, spinal cord or brain (11). Symptoms include loss of vision, eye pain, headaches, confusion, muscular stiffness or weakness, changes in bowel, bladder or sexual function, and seizures (11). Unlike MS, neurological deterioration in MOGAD does not usually progress without relapses, suggesting there is potential for effective treatment to reduce the accumulation of disability (1, 7).

The economic burden is likely to differ according to the nature of the clinical manifestation, the nerve region(s) impacted and the severity, number and frequency of attacks. Misdiagnoses and delayed treatment also play a role, as the heterogeneous presentations of MOGAD are not well recognised. The mean age of MOGAD onset is 28–30 years; however, approximately 30% of individuals with MOGAD are children (10). As a result, disability and sight loss can last for decades and the full economic impact is not known (1, 2).

There are currently no approved treatments for MOGAD. Clinical trials focused specifically on patients with MOGAD are currently ongoing. At present, treatment approaches to MOGAD in clinical practice are similar to those applied to other inflammatory demyelinating diseases. Case reports show that high-dose methylprednisolone (MP), intravenous immunoglobulin (IVIg) and plasma exchange are used to manage acute attacks, while methotrexate, azathioprine, corticosteroids, rituximab, IVIg and mycophenolate mofetil may be considered for maintenance therapy (2, 12, 13).

Given the current lack of evidence relating specifically to MOGAD, data from other antibody-mediated diseases where immunosuppression is commonly used to reduce inflammation and prevent relapse might provide greater insight. This decision was based on international recognition of extrapolation from related diseases as an acceptable approach to facilitate the approval of new medicines for rare diseases (14–17). We conducted a systematic literature search and here provide a narrative review of the economic impact of MOGAD. We included publications on autoimmune neurological conditions with similar clinical presentations or where similar treatments are used.

## Methods

A systematic literature search was designed to gather information on the health economic impacts of MOGAD and analogous conditions. This narrative review aligned with the Preferred Reporting Items for Systematic Reviews and Meta-Analysis (PRISMA) guidelines (18).

### Eligibility criteria

We selected any publication that presented the health economic impacts of MOGAD or analogous conditions as selected by the review team for inclusion (see author contributions for detail). Analogous conditions were selected based on comparison with MOGAD in terms of (a) clinical presentation, (b) treatments used in management, (c) pathophysiology and (d) incidence/prevalence; that is, they were autoimmune diseases of the nervous system with sufficient similarity in clinical presentation or management to MOGAD. Publications on MS were not included because of the higher prevalence and more mature treatment landscape of MS compared with MOGAD. Publications that did not specify a monetary amount but described predictors of cost (i.e., cost drivers) were eligible for inclusion. Exclusion criteria were as follows: non-English full text; lack of cost data for MOGAD or analogous condition (e.g., cost estimates spanning multiple neuroimmunological conditions); and data derived from cost modelling (e.g., Markov models) rather than true cost calculations. For hospitalisation costs, data relating to diagnostic costs alone were not included. Studies without annualised estimates were only included if drivers of cost were extractable.

### Information sources

The search was performed in PubMed on 14 July 2023 using medical subject headings (MeSH) and keywords for journals published between 2000 and 2022.

### Search strategy

The complete search strings, which include terms for cost analysis, economics, expenditure and expenses, are shown in Supplementary Table 1.

### Selection process

A reviewer (LL) undertook initial screening of the titles and abstracts; any uncertainty when applying the eligibility criteria was mediated by two other independent reviewers. The same reviewer (LL) then screened the full-text publications, with the references of included studies further assessed for inclusion eligibility. Data (cost estimates and driver data) were manually extracted after eligibility screening was completed.

### Data collection process and data items

Economic impacts included cost considerations from the patient or institutional (public or private) perspective, and costs associated with all stages of the patient journey. Diagnostic work-up, short-term interventions to aid recovery from an attack (e.g., hospitalisation, plasma exchange) and the management of long-term disability (e.g., carer costs, loss of income, support group services) were all considered. Reported costs were divided into direct costs, which denote those related to managing the condition in both the acute and the chronic setting, and indirect costs. The exhaustive list of considerations for direct costs included hospitalisation fees, post-discharge specialist fees, travel expenses for patients and caregivers, medication/diagnostic fees, at-home health services and support groups. For indirect costs, this study included morbidity (i.e., loss of productivity) and mortality (i.e., premature death from the illness). Mean annual costs per patient were extracted from each publication. Median costs were used where means were not available, and simple calculations were performed where needed to determine the annual cost per patient (e.g., when data were presented as monthly costs), and *p*-values for relevant comparisons were also extracted. No new statistical analyses were conducted. To ensure comparability, all reported costs were adjusted to 2024 US Dollars using the CCEMG-EPPI-Centre Cost Converter (19). The International Monetary Fund was used as the source dataset for purchasing power parities. If a cost year range was provided, the latter year was used for standardisation. If no cost year was provided, the publication year was used for standardisation. If the reporting country using Euros was not specified or if a pan-Europe study was conducted, Belgium was selected as the original study country. We referred to PRISMA in the reporting of our literature search, although owing to the range of conditions and study designs included, the literature search findings are summarised as a narrative review, and therefore not all PRISMA items were relevant.

## Results

### Literature search results

**Figure 1:**
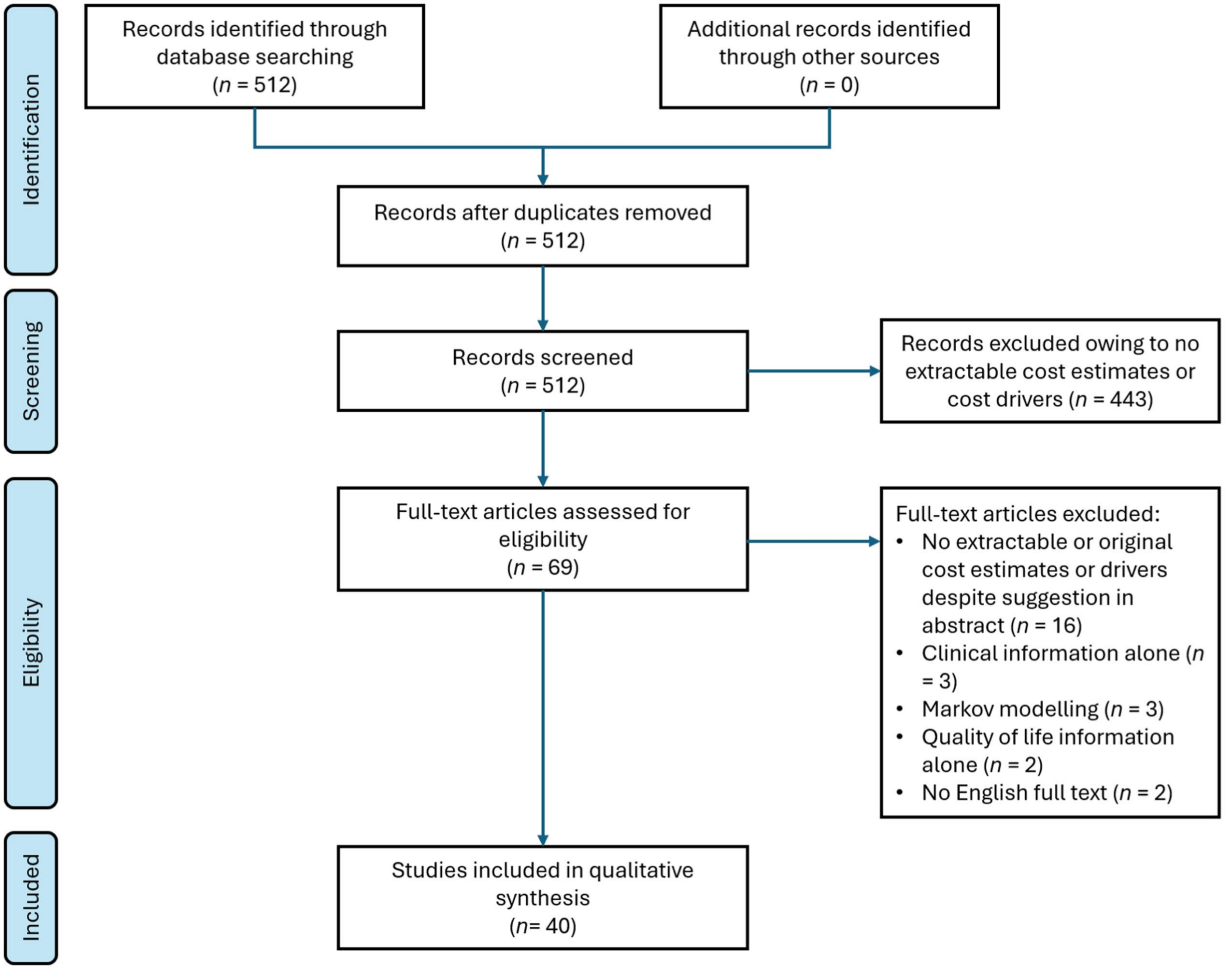
Flow chart of the systematic literature search in this study.

The search yielded 512 records, of which 443 were excluded during screening of the titles and abstracts. Of the remaining 69 records, 29 were excluded after full-text review with reasons presented in Figure 1. Results from the remaining 40 studies were extracted and tabulated.

**Figure 2:**
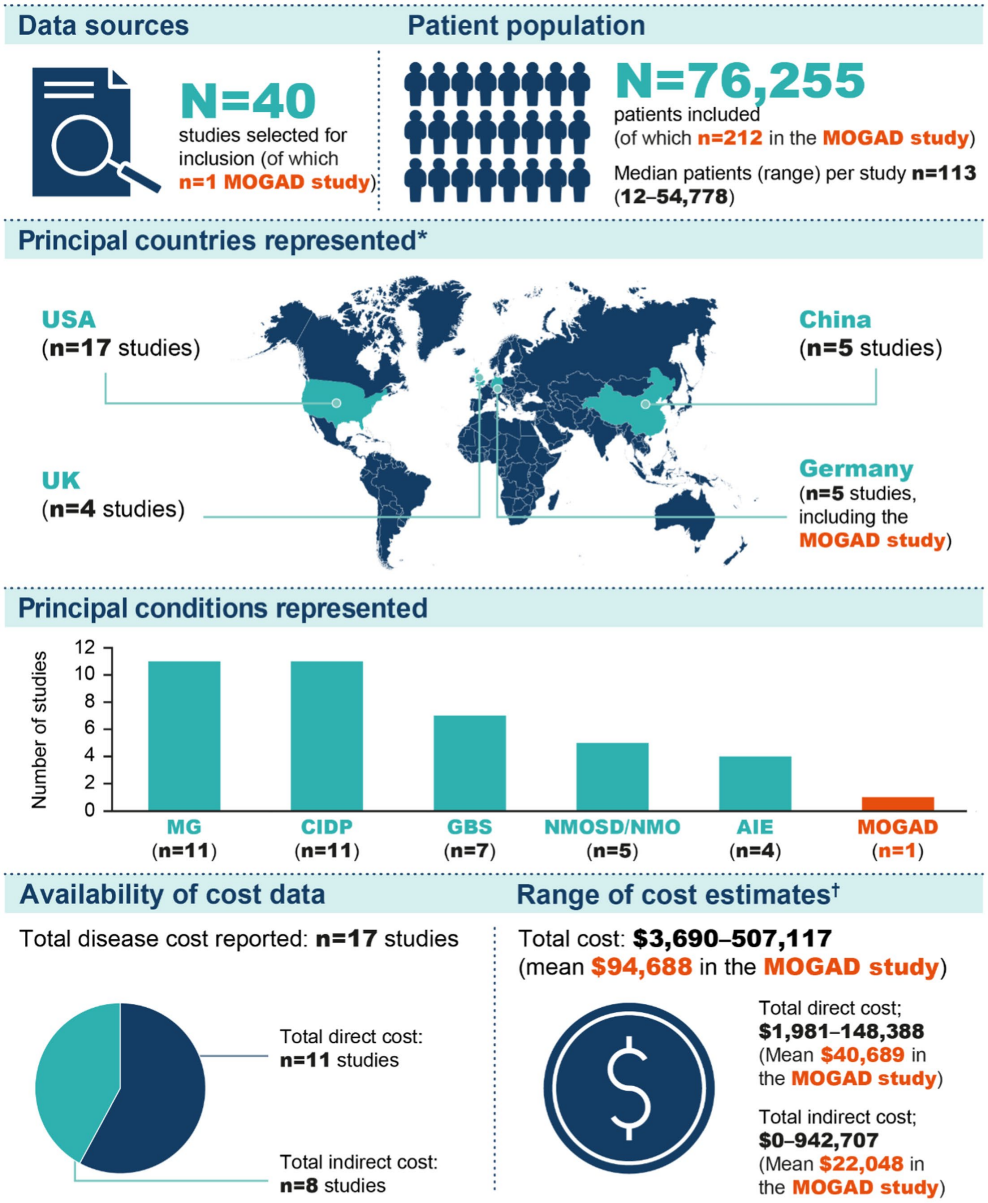
Summarised results of the literature search. *The numbers shown include one study conducted in Belgium, Czech Republic, Greece, Italy, Netherlands, Spain and the UK (52) and one study conducted on a global basis (40); all other studies were conducted in a single country. ^†^The three cost categories shown are not mutually exclusive (i.e., numerous studies contributed data to more than one category). AIE, autoimmune encephalitis; CIDP, chronic inflammatory demyelinating polyneuropathy; GBS, Guillain–Barré syndrome; MG, myasthenia gravis; MOGAD, myelin oligodendrocyte glycoprotein antibody-associated disease; NMO, neuromyelitis optica; NMOSD, neuromyelitis optica spectrum disorder.

Information on the methods, size and scope of the 40 included studies is provided in Figure 2 and Supplementary Table 2. In total, 76,255 patients were included, with all except four studies involving <2,000 patients (the four exceptions included 2,805, 3,341, 5,473 and 54,778 patients) (20–23). Most studies (*n* = 23) were cost analyses or cost-of-illness studies, four were cost minimisation analyses and there were four healthcare resource use studies. Fourteen of the studies were retrospective and 18 were non-comparative; there were four case–control studies but no randomised controlled trials. All studies except two were from a single country, and the countries with the largest numbers of studies were the USA, China, Germany and the UK. There were three studies each from India, Italy and the Netherlands, and additional countries in South America, Europe and Asia were represented by one or two studies only (the two international studies are included in these numbers). Most studies (*n* = 32) were from countries classified by the World Bank as high income; five were from countries with upper-middle incomes, two were from lower-middle income countries and one international study included countries of all four income levels (24).

**Figure 3:**
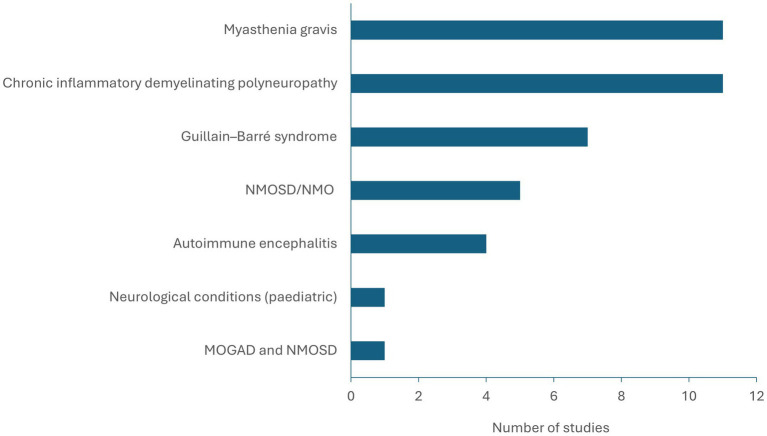
Representation of MOGAD and analogues within the literature (*n* = 40 studies). MOGAD, myelin oligodendrocyte glycoprotein antibody-associated disease; NMO, neuromyelitis optica; NMOSD, neuromyelitis optica spectrum disorder.

The conditions represented by the 40 included studies were chronic inflammatory demyelinating polyneuropathy (CIDP; *n* = 11), myasthenia gravis (MG; *n* = 11), Guillain–Barré syndrome (*n* = 7), NMOSD (*n* = 5) and autoimmune encephalitis (*n* = 4) (Figures 2, 3). MOGAD was described in only one study (25). Seventeen studies provided an estimate of the total cost of disease, and total direct and indirect costs were reported in 11 and 8 studies, respectively. One study was published in 2023, eight in 2022, three in 2021, six in 2020 and four in 2019. Fifteen studies were from 2010–2018, and the remaining three were published before 2010.

### Cost estimates

Tables 1–4 report cost estimates, grouped by disease. Studies reported different measures (i.e., not all studies reported total costs, direct costs or indirect costs) in different ways (most cost estimates were reported as means, but some were reported as medians; medians tended to be lower). The variability observed across the estimates can be attributed to the range of diseases and countries included in this review; there were generally not enough studies focusing on a given disease or from a given country to draw strong conclusions about specific diseases or countries.

**Table 1:** Summary of publications with extractable cost estimates and cost drivers: total costs.

Disease	Citation	Number of patients with the disease(s) of interest	Study type	Total costs*	Cost drivers	Additional notes
CIDP	Divino et al., 2018 (51)	790	Cost analysis (case–control study, retrospective)	[Cost of illness over 2 years] $148,399 (SD $228,494) in patients with CIDP; $19,883 ($72,321) in controls [Cost for CIDP therapy over 2 years] $76,055 (SD $174,630) in patients with CIDP; not applicable in controls	Medications, outpatient ancillary, radiology and inpatient care	In patients with CIDP, mean 2-year costs for ‘outpatient surgery’ and ‘outpatient ancillary, radiology and healthcare common procedure coding system drugs’ were $4,087 (SD $10,425) and $97,418 ($198,831), respectively*
CIDP	Mahdi-Rogers et al., 2014 (55)	43	Cost-of-illness study (patient survey, non-comparative)	[Cost of illness] $48,372 (95% CI: $32,576–$66,611)	NR	None
CIDP	McCrone et al., 2003 (52)	25	Cost–utility analysis (comparison of prednisolone vs. IVIg therapy)	[6-week total cost] $2,611 (SD $6,041) for patients treated with prednisolone and $9,456 ($1,121) for those treated with IVIg^†^	IVIg (more expensive than prednisolone)	None
CIDP	Mengel et al., 2018 (53)	108	Cost-of-illness study (patient survey, non-comparative)	[3-month total cost] $19,609 (95% CI: $16,626–$22,765)	IVIg (67% of the total cost) Reduced health-related quality of life and depressive symptoms (independent predictors for higher total costs)	None
Guillain–Barré syndrome	Frenzen, 2008 (20)	[Pan-US, patients hospitalised] 5,473	Cost analysis (non-comparative)	[Cost of illness, USA-wide] $2.776 billion (95% CI: $2.542–$3.010) Cost per patient: $507,117 (95% CI: $442,587–$571,647)	NR	None
Guillain–Barré syndrome	Oliveira et al., 2022 (58)	46	Cost-of-illness study (patient survey, non-comparative)	[Median cost of illness, from symptom onset until 6 months post-discharge, total period ~6.5 months] $1,999 (IQR: $892–$4,423)	NR	Costs shift during each section of the patient-care journey
Guillain–Barré syndrome	Tsai et al., 2007 (33)	24	Cost-effectiveness study (retrospective; includes comparison of IVIg vs. plasma exchange therapy)		Length of hospitalisation had a strong relationship with total cost (Pearson correlation coefficient 0.907) Total costs were higher for patients on ventilators than those not requiring ventilators (*p* = 0.008)	None
MOGAD^‡^ and NMOSD	Hümmert et al., 2022 (25)	212	Analysis of costs and HRQoL (patient survey, non-comparative)	[Cost of illness] $94,688 (95% CI: $81,418–$108,546)	Drivers: informal care (28% of total cost), indirect costs (23%) and medication (16%) Independent predictors: need for care, number of acute attacks, unemployment and disability measured by the Expanded Disability Scale Score Cost was positively correlated with disease severity, but neither antibody status nor disease duration influenced total cost	No cost difference between MOGAD and NMOSD stratified by serostatus and disease duration was found, apart from higher outpatient diagnostic test for MOGAD
Myasthenia gravis	Fan et al., 2020 (59)	69	HRQoL study (cross-sectional, non-comparative)	Patients paid a median of $180 per month to ease problems related to myasthenia gravis, despite medical insurance coverage	NR	Financial burden (ratio of myasthenia gravis expenditure to total income) was negatively associated with total SF-36 score (*p* < 0.01)
Myasthenia gravis	Guptill et al., 2012 (60)	113	Cost analysis (includes comparison of costs for IVIg vs. plasma exchange therapy)	[Cost of illness] $27,234 (95% CI: $14,629–$39,840), compared with $6,090 ($4,880–$7,300) in matched controls (*p* < 0.001)	NR	Home health costs were significantly greater in patients with myasthenia gravis than in controls
Myasthenia gravis	Harris et al., 2020 (56)	782	Healthcare resource utilisation study (retrospective, non-comparative)	NR	Refractory disease	Refractory myasthenia gravis was correlated to worse activities of daily living and increased healthcare resource use
Myasthenia gravis	Ignatova et al., 2022 (27)	54	Cost-of-illness study (patient survey, non-comparative)	[Median] $5,870 (IQR: $1,250–$13,843)^†,§^	Disease severity, relapses (significant predictors of cost)	None
Myasthenia gravis	Schepelmann et al., 2010 (54)	107 patients with amyotrophic lateral sclerosis (*n* = 46), myasthenia gravis (*n* = 41) or facioscapulohumeral muscular dystrophy (*n* = 20)	Patient questionnaires and diaries (non-comparative)	[Total annual cost of myasthenia gravis from societal perspective] $27,231 (95% CI: $19,071–$39,851)	Considering all 3 diseases of the study, the main components of costs were the expenditures of health insurance and the loss of productivity of patients and their caregivers. For myasthenia gravis, disease severity and assistance in ADL were identified as independent cost-driving factors	[For patients with all 3 diseases of the study – amyotrophic lateral sclerosis, myasthenia gravis and facioscapulohumeral muscular dystrophy] Average total cost due to comorbidities unrelated to neuromuscular disease was $10,856
Myasthenia gravis	Sonkar et al., 2017 (30)	66	Cost analysis (prospective, non-comparative)	[Median, *n* = 66] $4,449 (range: $330–$38,569)	Severity of disease and treatment with IVIg or plasma exchange were identified as determinants of cost	The predictors of cost were severe myasthenia gravis (*r* = 0.43; *p* < 0.0001), myasthenic crisis (*r* = 0.54; *p* = 0.0001), mechanical ventilation (*r* = 0.36; *p* = 0.003) and hospitalisation or ICU admission (*r* = 0.54; *p* = 0.0001)
Myasthenia gravis	Ting et al., 2023 (61)	1,498	Analysis of healthcare resource utilisation and costs (retrospective study of insurance claims data, non-comparative)	Among patients with myasthenia gravis, the mean all-cause total healthcare cost was $128,288 per patient during 2 years of follow-up, with $105,733 and $22,554 attributed to medical and pharmacy costs, respectively	NR	None
NMOSD	Hughes et al., 2022 (36)	117 patients and 74 informal carers	Analysis of health utilities and costs (patient survey, non-comparative)	[Total patient cost per patient during 3 months preceding questionnaire completion] $10,295 (95% central range: $3,837–$22,256)	Extent of disability	Patient costs (self-reported, including private medication and house adjustment costs) during the 3 months preceding questionnaire completion were $1,289 (95% central range: $397–$2,766)

*Cost data were standardised according to the cost year stated in the reference manuscript. If a cost year range was provided, the latest date was taken. If no cost year was provided, the cost year was set to the publication year. ^†^If the reporting country using Euros was not specified or if a pan-Europe study was conducted, Belgium was selected. ^‡^Patients with MOGAD diagnosed according to Jarius et al. (34). ^§^Costs are reported in Euros with no information pertaining to the conversion from local currency at the time of analysis. Standardised costs (USD) shown are mean annual cost per patient unless otherwise indicated. The study by Mahdi-Rogers et al. (55) included three different diseases (CIDP, multifocal motor neuropathy and paraproteinaemic demyelinating neuropathy), but here we have extracted data for CIDP only. The study by Schepelmann et al. (54) included three different diseases (amyotrophic lateral sclerosis, facioscapulohumeral muscular dystrophy and myasthenia gravis), but here we have extracted data for myasthenia gravis only. Please see Supplementary Table 2 for further details of the studies included in this table. ADL, activities of daily living; CI, confidence interval; CIDP, chronic inflammatory demyelinating polyneuropathy; HRQoL, health-related quality of life; ICU, intensive care unit; IQR, interquartile range; IVIg, intravenous immunoglobulin; MOGAD, myelin oligodendrocyte glycoprotein antibody-associated disease; NMOSD, neuromyelitis optica spectrum disorder; SD, standard deviation; USD, US dollars.

**Table 2:** Summary of publications with extractable cost estimates: direct costs.

Disease	Citation	Number of patients with the disease(s) of interest	Study type	Total direct costs*	Medical or healthcare costs*	Medication costs*	Immunoglobulin costs*	Hospital costs*	Outpatient healthcare costs*
Autoimmune encephalitis	Cai et al., 2022 (62)	78	Cost-efficiency analysis (retrospective, comparison of IV methylprednisolone monotherapy vs IV methylprednisolone plus IVIg combination therapy)	NR	NR	NR	IVIg monotherapy $13,729 (IQR: $10,585–$15,409)	NR	NR
Autoimmune encephalitis	Cohen et al., 2019 (26)	63	Cost analysis (retrospective; includes comparison of antibody-positive vs. antibody-negative patients)	NR	NR	NR	NR	[Median] $93,039 (IQR: $47,032–$216,602) overall; $109,187 ($57,282–$267,222) for antibody-positive patients vs. $70,721 ($40,480–$194,808) for antibody-negative patients (*p* = 0.21)	NR
Autoimmune encephalitis	Li et al., 2020 (63)	208	Cost analysis (retrospective, non-comparative)	$29,191 (SD $28,973)	[Medical cost] $27,406 (SD $27,262)	[Treatment cost] $14,525 (SD $11,050)	[Immunotherapy cost] $11,852 (SD $8,743)	[Inpatient cost] $26,921 (SD $27,141)	$485 (SD $1,353)
Autoimmune encephalitis	Sharp et al., 2021 (64)	58 patients in 2018 and 53 patients during the first 7 months of 2019	Cost analysis (relating to autoimmune encephalopathy testing panels; costs compared before and after implementing an algorithm for ordering panels)	NR	NR	NR	NR	NR	NR
CIDP	Allen et al., 2020 (65)	289	Hypothetical cost analysis (reported within a review article; includes comparison of low-dose SCIg, high-dose SCIg and IVIg)	NR	NR	NR	Low-dose SCIg $9,906; high-dose SCIg $19,790; comparative cost for IVIg $10,966 (applicable to both high- and low-dose settings)	NR	NR
CIDP	Darbà & Marsà, 2022 (23)	2,805	Cost analysis [retrospective; includes comparison of two different time periods (2004–2015 vs. 2015–2016)]	NR	NR	NR	NR	[Mean medical cost per hospital admission] $7,521 ($6,531 for ≤4 days and $8,740 for >4 days)	$7,821
CIDP	Divino et al., 2018 (51)	790	Cost analysis (case-control study, retrospective)	NR	[Direct medical costs over 2 years, inclusive of outpatient pharmacy and medical claims] $76,055 (SD $174,630) (mean value represents 51.2% of mean total cost)	NR	NR	[Inpatient care over 2 years] $20,866 (SD $84,861) in patients with CIDP; $3,651 ($22,014) in controls	NR
CIDP	Guptill et al., 2014 (28)	73	Analysis of costs and healthcare resource use (retrospective, non-comparative)	[Health plan paid cost] $74,116 (95% CI: $47,443–$100,789)	[Medical cost] $32,604 (95% CI: $14,860–$50,349)	NR	IVIg $38,022 overall (90% of overall pharmacy cost) and $140,566 per patient (SE $23,993) who received this treatment	NR	NR
CIDP	Le Masson et al., 2018 (66)	24	Cost minimisation analysis (before and after analysis with prospective data collection; comparison of home- vs. hospital-administered IVIg)	[Total treatment cost] $73,696 (SD $39,923) with home treatment and $140,388 ($78,186) with hospital treatment	NR	NR	[For patients receiving hospital IVIg] $6,882 per year (SD $2,143) [For patients receiving home IVIg] $7,608 (SD $2,461) per year, plus additional cost of $754 for nursing, dispensing, infusion pump, pump supplies and IVIg transportation	[For patients receiving hospital IVIg] $10,480 (SD $5,264) [For patients receiving home IVIg] $820 (SD $335)	NR
CIDP	Mahdi-Rogers et al., 2014 (55)	43	Cost-of-illness study (patient survey, non-comparative)	$35,636 (95% CI: $22,278–$51,269)	Estimated UK-wide annual treatment cost: $38,304,679	NR	IVIg: $22,664	$6,358 (SD $9,446) Estimated UK-wide cost $10,611,940	NR
CIDP	McCrone et al., 2003 (52)	25	Cost–utility analysis (comparison of prednisolone vs. IVIg therapy)	NR	NR	NR	IVIg $8,600 (SD $0)^†^	[6-week inpatient cost] $1,849 (SD $4,980) for patients treated with prednisolone and $0 ($0) for those treated with IVIg^†^	[6-week outpatient cost] $123 (SD $293) for patients treated with prednisolone and $257 ($605) for those treated with IVIg^†^
CIDP	Mengel et al., 2018 (53)	108	Cost-of-illness study (patient survey, non-comparative)	[3-month direct cost] $16,304 (95% CI: $13,756–$18,947) (mean represents 83% of the mean total cost)	NR	[3-month cost for all drugs] $13,664 (95% CI: $11,344–$16,092)	3-month cost of IVIg $13,161 (95% CI: $9,101–$15,602)	[3-month cost for inpatient care, based on a rate of $848 per patient per day] $1,248 (95% CI: $801–$1,729)	[3-month cost] $265 (95% CI: $232–$298)
CIDP	Perraudin et al., 2020 (67)	Not applicable (modelling study)	Cost minimalisation analysis (comparison of hospital-based IVIg vs. home-based SCIg)	NR	NR	NR	[Estimated total costs for treatment over 48 weeks] Hospital-based IVIg $105,236; home-based SCIg $80,671	NR	NR
CIDP	Piscitelli et al., 2021 (68)	12	Cost analysis (retrospective; comparison of SCIg vs. IVIg)	NR	NR	NR	[Estimated total costs for treatment of 12 patients over 1 year] IVIg $588,059; SCIg $1,000,087	NR	NR
CIDP	Rajabally & Afzal, 2019 (69)	39	Retrospective study (review of hospital patient records; comparison of different dosing methods for IVIg)	NR	NR	NR	[IVIg] Cost when administered according to an individualised, outcome-measured, dose-modifying protocol $98,573 (drug cost $67,773, infusion-related cost $30,800) Corresponding total values for standard dosing by dosing weight and standard dosing by recorded weight $110,281 and $122,879, respectively	NR	NR
Guillain–Barré syndrome	Frenzen, 2008 (20)	[Pan-US, patients hospitalised] 5,473	Cost analysis (non-comparative)	[Direct medical cost, USA-wide] $394 million (95% CI: $337 million – $452 million) (point estimate represents 14% of the total cost of illness) [Direct medical cost per patient] $72,023 ($59,557–$84,489)	[Direct medical cost, USA-wide] $394 million (95% CI: $337 million – $452 million) [Direct medical cost, per patient] $72,023 ($59,557–$84,489)	NR	NR	NR	NR
Guillain–Barré syndrome	Maheshwari et al., 2018 (70)	40	Cost minimisation analysis (includes comparison of IVIg vs. plasmapheresis therapy)	NR	[Annual health system cost] mean $514 per patient treated (total cost of $228,128 incurred for 4,441 patients)	NR	Out-of-pocket cost for IVIg: $5,698	NR	NR
Guillain–Barré syndrome	Oliveira et al., 2022 (58)	46	Cost minimisation analysis (includes comparison of IVIg vs. plasmapheresis therapy)	[Median total direct cost, from symptom onset until 6 months post-discharge, total period ~6.5 months] $1,286 (IQR: $221–$2,215) (median value represents 58% of the median total cost of illness)	[Median direct medical cost, from symptom onset until 6 months post-discharge, total period ~6.5 months] $232 (IQR: $35–$698)	[Median cost of medication] From symptom onset to admission $0 For the 6-month period following discharge $23 (IQR: $0–$127)	NR	NR	NR
Guillain–Barré syndrome	Rumalla et al., 2017 (22)	54,778	Retrospective study (review of data from the US Nationwide Inpatient Sample, comparison of patients with Guillain–Barré syndrome with vs. without hyponatraemia)	NR	NR	NR	NR	[Total hospital cost] $74,204 (SD $70,452) for patients with Guillain–Barré syndrome with hyponatraemia, vs. $46,892 ($57,491) for patients with Guillain–Barré syndrome without hyponatraemia (*p* < 0.0001)	NR
Guillain–Barré syndrome	Tsai et al., 2007 (33)	24	Cost-effectiveness study (retrospective; includes comparison of IVIg vs. plasma exchange therapy)	NR	NR	NR	Except for the costs of the drugs used in IVIg, treatment of Guillain–Barré syndrome with IVIg was more cost-effective (*p* = 0.057) than with plasma exchange in total length of hospitalisation and the cost of procedures and hospitalisation	Each additional day of hospitalisation increased costs by $456	NR
Guillain–Barré syndrome	van Leeuwen et al., 2016 (71)	87	Analysis of resource use and costs (non-comparative)	NR	NR	NR	NR	[Median cost for hospital admission] $24,554 (IQR: $18,303–$38,612)	NR
Guillain–Barré syndrome	Winters et al., 2011 (31)	Not applicable (modelling study)	Cost minimisation analysis (comparison of IVIg vs. plasma exchange)	NR	NR	NR	IVIg (5 infusions) $14,188	NR	NR
MOGAD^‡^ and NMOSD	Hümmert et al., 2022 (25)	212	Analysis of costs and HRQoL (patient survey, non-comparative)	$72,640 (77% of the total cost)	[Direct medical cost] $40,689 (95% CI: $36,129–$68,091) (mean value represents 43% of the mean total cost)	[Including apheresis] $15,554 (95% CI: $12,560–$19,149)	[Immunotherapy] $12,329 (95% CI: $9,729–$15,921)	[Inpatient hospital care] $8,263 (95% CI: $6,205–$10,514)	[Outpatient consultations] $941 (95% CI: $734–$1,175)
Myasthenia gravis	Fan et al., 2020 (59)	69	HRQoL study (cross-sectional, non-comparative)	NR	NR	NR	NR	NR	NR
Myasthenia gravis	Guptill et al., 2011 (29)	1,288	Cost analysis (includes comparison of costs for IVIg vs. plasma exchange therapy)	NR	NR	[Average claims-based cost per patient with myasthenia gravis] $34,337 (SD $66,244) [Total US pharmacy cost related to myasthenia gravis] $12.9 million	US cost for IVIg $11.0 million (85% of pharmacy cost) Mean cost per IVIg infusion $6,408 (SD $7,287)	US cost for hospital-based treatment of patients with myasthenia gravis $11,702,970	NR
Myasthenia gravis	Guptill et al., 2012 (60)	113	Cost analysis (case-control study)	NR	NR	NR	NR	NR	NR
Myasthenia gravis	Harris et al., 2020 (56)	782	Healthcare resource utilisation study (retrospective, non-comparative)	NR	NR	NR	NR	NR	NR
Myasthenia gravis	Ignatova et al., 2022 (27)	54	Cost-of-illness study (patient survey, non-comparative)	[Median] $1,981 (IQR: $1,149–$7,651)^†^	NR	[Median for drugs] $1,118 (IQR: $643–$1,131)^†^	NR	[Median] $0^†^	NR
Myasthenia gravis	Mandawat et al., 2010 (32)	1,606 (908 patients with myasthenia gravis and 698 patients with myasthenia gravis crisis)	Economic outcomes study (retrospective, comparison of patients with myasthenia gravis with vs. without crisis)	NR	NR	NR	NR	[Patients with myasthenia gravis] Inpatient cost was $37,398 (IQR: $35,011) for patients receiving plasma exchange vs. $29,630 ($29,382) for those receiving IVIg (*p* < 0.001) [Patients with myasthenia gravis with crisis] Inpatient cost was $75,466 (IQR: $91,644) for patients receiving plasma exchange vs. $47,584 ($48,869) for those receiving IVIg (*p* < 0.0001)	NR
Myasthenia gravis	Qi et al., 2022 (72)	1,225	IVIg utilisation study (retrospective, comparison of chronic vs. intermittent users of IVIg)	NR	[Medical cost] $197,408 for patients using IVIg chronically vs. $79,326 for those with intermittent IVIg use (*p* < 0.001)	NR	NR	NR	NR
Myasthenia gravis	Schepelmann et al., 2010 (54)	107 patients with amyotrophic lateral sclerosis (*n* = 46), myasthenia gravis (*n* = 41) or facioscapulohumeral muscular dystrophy (*n* = 20)	Patient questionnaires and diaries (non-comparative)	[From societal perspective] $21,566 (95% CI: $15,063–$33,697)	NR	[Drugs] $3,279 (95% CI: $2,732–$4,299)	[Immuno-modulation] $1,093 (95% CI: $510–$1,348)	$15,264 (95% CI: $8,980–$26,921)	$2,095 (95% CI: $1,676–$2,605)
Myasthenia gravis	Sonkar et al., 2017 (30)	66	Cost analysis (prospective, non-comparative)	[Median, *n* = 66] $3,322 (range: $330–$32,482)	NR	NR	NR	[Median, *n* = 50] $3,232 (range: $269–$31,146)	[Median, *n* = 16] $1,481 (range $330–$4,271)
Myasthenia gravis	Ting et al., 2023 (61)	1,498	Analysis of healthcare resource utilisation and costs (retrospective study of insurance claims data, non-comparative)	NR	[Mean total myasthenia gravis-related medical cost over 2 years] $71,245 [Mean total myasthenia gravis-related healthcare cost over 2 years] $83,114	NR	[Mean IVIg-specific outpatient cost over 2 years] Whole cohort (*n* = 1,498) $22,953, patients on chronic IVIg (*n* = 114) $163,430 [Mean IVIg-specific inpatient cost over 2 years] Whole cohort (*n* = 1,498) $2,364, patients on chronic IVIg (*n* = 114) $1,986	[Mean total myasthenia gravis-related inpatient cost over 2 years] $24,614 [Mean total myasthenia gravis-related inpatient treatment cost over 2 years] $5,720	[Mean total myasthenia gravis-related outpatient cost over 2 years] $46,631 [Mean total myasthenia gravis-related outpatient treatment cost over 2 years] $27,338
Neuro-immunological conditions (paediatric)	Nosadini et al., 2016 (73)	196	Retrospective study (chart review; includes comparison of immunoglobulin costs for patients with different neuroimmunological conditions)	NR	NR	NR	[IVIg cost per patient over the 14-year study period] Overall cohort (*n* = 196) $17,064 (range $701–$335,986), monophasic inflammatory demyelinating CNS diseases (*n* = 22) $3,630, relapsing inflammatory demyelinating CNS diseases (*n* = 7) $25,233, acute demyelinating neuropathy (Guillain–Barré syndrome) (*n* = 55) $4,337, chronic demyelinating neuropathies (*n* = 9) $81,209, myasthenia gravis (*n* = 12) $23,802	NR	NR
NMOSD	Beekman et al., 2019 (38)	193	Cost analysis (performed within a study of patient experience and quality of life, non-comparative)	NR	NR	[Prescription medications] $2,253	NR	$8,705	NR
NMOSD	Exuzides et al., 2021 (37)	162	Cost analysis (case-control study)	NR	NR	NR	[Median cost of IVIg among patients claiming for this treatment] $29,247 (IQR: $6,515–$51,611)	$34,893 (SD $173,986) in patients with NMOSD; $1,827 ($12,921) in non-NMOSD controls	$29,881 (SD $42,590) in patients with NMOSD; $5,718 ($31,762) in non-NMOSD controls
NMO	Holroyd et al., 2019 (40)	60 physicians	Analysis of availability and affordability of neuromyelitis optica testing and treatment (physician survey, non-comparative)	NR	[Treatment] $5,248	NR	NR	NR	NR
NMOSD	Hughes et al., 2022 (36)	117 patients and 74 informal carers	Analysis of health utilities and costs (patient survey, non-comparative)	NR	NR	[During the 3 months preceding questionnaire completion] $1,111 (95% central range: $381–$2,671)	NR	[Inpatient stays during the 3 months preceding questionnaire completion] $7,239 (95% central range: $932–$16,882)	$582 (95% central range: $449–$769)
NMOSD	Knapp et al., 2022 (39)	130	Cost analysis (based on health insurance data, comparison of patients with active disease vs. inactive disease vs. controls)	NR	[Healthcare cost] $18,189 in patients with NMOSD vs. $6,575 in non-NMOSD controls (*p* < 0.001)	NR	NR	$9,083 in patients with NMOSD vs. $2,730 in non-NMOSD controls (*p* < 0.001)	[Outpatient care costs] $1,241 in patients with NMOSD vs. $1,171 in non-NMOSD controls (*p* < 0.001) [Outpatient prescription costs] $4,699 in patients with NMOSD vs. $1,462 in non-NMOSD controls

*Cost data were standardised according to the cost year stated in the reference manuscript. If a cost year range was provided, the latest date was taken. If no cost year was provided, the cost year was set to the publication year. ^†^If the reporting country using Euros was not specified or if a pan-Europe study was conducted, Belgium was selected. ^‡^Patients with MOGAD diagnosed according to Jarius et al. (34). Costs shown are mean annual cost per patient unless otherwise indicated. Study by Mahdi-Rogers et al. (55) included three different diseases (CIDP, multifocal motor neuropathy and paraproteinaemic demyelinating neuropathy), but here we have extracted data for CIDP only. The study by Schepelmann et al. (54) included three different diseases (amyotrophic lateral sclerosis, facioscapulohumeral muscular dystrophy and myasthenia gravis), but here we have extracted data for myasthenia gravis only. The publication by Allen et al. (65) is a review, retained because cost data therein appear to be original. Data inconsistencies were encountered in the publication by Winters et al. (31); for these, values from tables within the publication, rather than the main text, were selected for inclusion here. Please see Supplementary Table 2 for further details of the studies included in this table. CI, confidence interval; CIDP, chronic inflammatory demyelinating polyneuropathy; CNS, central nervous system; HRQoL, health-related quality of life; IgG, immunoglobulin G; IQR, interquartile range; IV, intravenous; IVIg, intravenous immunoglobulin; MOGAD, myelin oligodendrocyte glycoprotein antibody-associated disease; NMO, neuromyelitis optica; NMOSD, neuromyelitis optica spectrum disorder; NR, not reported; SCIg, subcutaneous immunoglobulin; SD, standard deviation.

**Table 3:** Summary of publications with extractable cost drivers: direct costs.

Disease	Citation	Number of patients with the disease(s) of interest	Study type	Hospitalisation rate	Direct cost drivers*	Additional notes*
Autoimmune encephalitis	Cai et al., 2022 (62)	78	Cost-efficiency analysis (retrospective, comparison of IV methylprednisolone monotherapy vs. IV methylprednisolone plus IVIg combination therapy)	NR	NR	Costs for IVMP monotherapy and combination therapy with IVMP + IVIg were $3,251 (IQR: $2,916–$8,264) and $13,939 ($11,781–$18,411), respectively
Autoimmune encephalitis	Cohen et al., 2019 (26)	63	Cost analysis (retrospective; includes comparison of antibody-positive vs. antibody-negative patients)	[Study conducted in a population of inpatients] Median length of stay was 15 days and 27 patients (43%) were admitted to the ICU	NR	No difference in hospital cost between patients with cell surface or intracellular antibody autoimmune encephalitis. Compared with patients with herpes simplex encephalitis, those with autoimmune encephalitis had a longer hospital stay (median 3× longer) and longer stay in ICU (2×). Contributors to longer hospital stay and thus cost were treatment (specifically plasmapheresis lasting ≥7 days), delays in establishing diagnosis and refractoriness to first-line treatment
Autoimmune encephalitis	Li et al., 2020 (63)	208	Cost analysis (retrospective, non-comparative)	NR	Length of stay in hospital	Mean direct non-medical cost was $1,785 (SD $2,009). Cost of LGI1/CASPR2 autoimmune encephalitis was significantly less than that of NMDA/GABA_B_ autoimmune encephalitis
Autoimmune encephalitis	Sharp et al., 2021 (64)	58 patients in 2018 and 53 patients during the first 7 months of 2019	Cost analysis (relating to autoimmune encephalopathy testing panels; costs compared before and after implementing an algorithm for ordering panels)	NR	NR	[2018] Cost for ordering 77 autoimmune encephalitis testing panels (for use in 58 patients) was $168,107 [2019, before implementing an algorithm for ordering panels] Based on data from the first 7 months, cost for ordering 95 panels for the whole year was estimated to exceed $216,174 [Effect of implementing the algorithm] There was a 43% decrease in the total number of panels ordered, the true-positive rate increased >3-fold and estimated annual cost saving was $30,024
CIDP	Allen et al., 2020 (65)	289	Hypothetical cost analysis (reported within a review article; includes comparison of low-dose SCIg, high-dose SCIg and IVIg)	NR	NR	None
CIDP	Darbà & Marsà, 2022 (23)	2,805	Cost analysis (retrospective; includes comparison of two different time periods (2004–2015 vs. 2015–2016))	Hospitalisation rate in 2018 was 12.1 per 100,000 patients. Mean length of hospital stay 8.1 days (95% CI: 7.7–8.5 days)	NR	The hospitalisation rate increased significantly between 2004 and 2015 and decreased between 2015 and 2016, coinciding with implementation of new ICD coding
CIDP	Divino et al., 2018 (51)	790	Cost analysis (case-control study, retrospective)	NR	NR	None
CIDP	Guptill et al., 2014 (28)	73	Analysis of costs and healthcare resource use (retrospective, non-comparative)	NR	The mean pharmacy cost was $41,512 (95% CI: $22,379–$60,643) (mean value represents 57% of the mean total health plan cost)	None
CIDP	Le Masson et al., 2018 (66)	24	Cost minimisation analysis (before and after analysis with prospective data collection; comparison of home- vs. hospital-administered IVIg)	[For patients receiving hospital IVIg] Mean 2.8 hospital days per year (SD 1.1 days) [For patients receiving home IVIg] Mean 0.08 (SD 0.5) hospital days per year	NR	None
CIDP	Mahdi-Rogers et al., 2014 (55)	43	Cost-of-illness study (patient survey, non-comparative)	[6-month usage data] Outpatient services, 62.8% of patients; inpatient or day-case services, 39.5%	The cost of IVIg was the major component of the total healthcare cost	None
CIDP	McCrone et al., 2003 (52)	25	Cost–utility analysis (comparison of prednisolone vs. IVIg therapy)	NR	NR	None
CIDP	Mengel et al., 2018 (53)	108	Cost-of-illness study (patient survey, non-comparative)	NR	Medication costs (detail not reported)	The 3-month direct cost of $16,304 (95% CI: $13,756–$18,947) consisted of $15,479 ($12,965–$18,085) of health insurance costs and $825 ($204–$1,737) of out-of-pocket costs
CIDP	Perraudin et al., 2020 (67)	Not applicable (modelling study)	Cost minimalisation analysis (comparison of hospital-based IVIg vs. home-based SCIg)	NR	NR	In this comparison of SCIg vs. IVIg, IgG was the major cost driver
CIDP	Piscitelli et al., 2021 (68)	12	Cost analysis (retrospective; comparison of SCIg vs. IVIg)	NR	NR	This was a comparison of SCIg vs. IVIg
CIDP	Rajabally & Afzal, 2019 (69)	39	Retrospective study (review of hospital patient records; comparison of different dosing methods for IVIg)	NR	NR	This was a comparison of different dosing methods for IVIg
Guillain–Barré syndrome	Frenzen, 2008 (20)	[Pan-US, patients hospitalised] 5,473	Cost analysis (non-comparative)	Annual number of hospitalisations (community hospitals) in the USA due to Guillain–Barré syndrome: 6,008 (95% CI: 5,510–6,506)	Community hospital admissions	USA-wide annual outpatient care due to Guillain–Barré syndrome: 19,728 physician visits (95% CI: 0–103,506), 147,182 physical therapy visits (0–309,820) and 7,821 occupational therapy visits (0–29,553)
Guillain–Barré syndrome	Maheshwari et al., 2018 (70)	40	Cost minimisation analysis (includes comparison of IVIg vs. plasmapheresis therapy)	For patients admitted to a tertiary care hospital, average length of stay in ward was 7.4 days	NR	Out-of-pocket cost for IVIg was higher ($5,698) than for plasmapheresis ($2,704), but the clinical efficacy of these treatments was the same
Guillain–Barré syndrome	Oliveira et al., 2022 (58)	46	Cost minimisation analysis (includes comparison of IVIg vs. plasmapheresis therapy)	NR	NR	Non-medical costs accounted for 57.9% of the total direct cost Within direct non-medical expenses, travel was the highest cost during symptom onset/hospitalisation, which changes through the 6-month post-discharge period
Guillain–Barré syndrome	Rumalla et al., 2017 (22)	54,778	Retrospective study (review of data from the US Nationwide Inpatient Sample, comparison of patients with Guillain–Barré syndrome with vs. without hyponatraemia)	Mean length of stay 16.07 days (SD 15.40 days) for Guillain–Barré patients with hyponatraemia, vs. 10.41 days (13.73) for Guillain–Barré patients without hyponatraemia (*p* < 0.0001)	NR	Incidence of hyponatraemia in this cohort of patients with Guillain–Barré syndrome was 11.8%
Guillain–Barré syndrome	Tsai et al., 2007 (33)	24	Cost-effectiveness study (retrospective; includes comparison of IVIg vs. plasma exchange therapy)	NR	Ventilator requirement	None
Guillain–Barré syndrome	van Leeuwen et al., 2016 (71)	87	Analysis of resource use and costs (non-comparative)	Median hospital stay was 17 days (IQR: 11–26 days) (all study participants were admitted to hospital)	NR	This was a hospital-based study. Hospital admission cost was highly associated with disease severity (measured by maximal Guillain–Barré syndrome disability score), ranging from $3,959 (IQR: $1,298–$6,205) for patients with a score of 1 to $96,465 ($73,418–$111,468) for patients with a score of 5
Guillain–Barré syndrome	Winters et al., 2011 (31)	Not applicable (modelling study)	Cost minimisation analysis (comparison of IVIg vs. plasma exchange)	NR	NR	This study was performed to compare direct costs of IVIg and plasma exchange Cost of plasmapheresis (5 procedures): $6,373
MOGAD^‡^ and NMOSD	Hümmert et al., 2022 (25)	212	Analysis of costs and HRQoL (patient survey, non-comparative)	NR	Direct medical costs driven by medication (38% of direct medical costs), then inpatient hospital care costs (20%), and formal care costs ($5,840 [95% CI: $2,872–$10,161], 14%) Direct non-medical costs driven by informal care ($26,162 [95% CI: $21,041–$31,590], 82% of the total direct non-medical cost) Direct medical and non-medical costs increased with disease severity	Direct non-medical cost was $31,951 (95% CI: $25,052–$39,138) (mean value represents 34% of the mean total cost)
Myasthenia gravis	Fan et al., 2020 (59)	69	HRQoL study (cross-sectional, non-comparative)	NR	NR	73.9% of the patients consulted a doctor >12 times per year
Myasthenia gravis	Guptill et al., 2011 (29)	1,288	Cost analysis (includes comparison of costs for IVIg vs. plasma exchange therapy)	NR	NR	Mean cost per plasma exchange session (for comparison with the IVIg infusion cost) was $1,304 (SD $1,608) US costs for non-steroidal immunosuppressives (9.3% of total pharmacy cost), cholinesterase inhibitors (5.7%) and corticosteroids (0.2%) were $1,201,264, $736,258 and $258,336, respectively
Myasthenia gravis	Guptill et al., 2012 (60)	113	Cost analysis (case-control study)	Among the 113 patients, 14 had a total of 38 inpatient hospital admissions, and 83 had a total of 578 outpatient hospital visits during the analysis year	The mean annual pharmacy cost, including outpatient but not inpatient IVIg, was $12,156 (95% CI: $2,303–$22,010) for patients with myasthenia gravis and $820 ($646–$994) for controls (*p* < 0.001)	None
Myasthenia gravis	Harris et al., 2020 (56)	782	Healthcare resource utilisation study (retrospective, non-comparative)	6-month probability of hospitalisation decreased over time, from ~20–30% at baseline to ~15–20% at 4 years	NR	None
Myasthenia gravis	Ignatova et al., 2022 (27)	54	Cost-of-illness study (patient survey, non-comparative)	NR	Medications	None
Myasthenia gravis	Mandawat et al., 2010 (32)	1,606 (908 patients with myasthenia gravis and 698 patients with myasthenia gravis crisis)	Economic outcomes study (retrospective, comparison of patients with myasthenia gravis with vs. without crisis)	[Patients with myasthenia gravis without crisis] Length of stay 6 days (IQR: 5 days) for patients receiving plasma exchange vs. 4 days (3 days) for those receiving IVIg (*p* < 0.001) [Patients with myasthenia gravis crisis] Length of stay 10 days (IQR: 11 days) for patients receiving plasma exchange vs. 5 days (5 days) for those receiving IVIg (*p* < 0.0001)	NR	The rate of clinical complications was 30.1% in patients with myasthenia gravis crisis on plasmapheresis and 14.8% in those on IVIg (*p* < 0.001)
Myasthenia gravis	Qi et al., 2022 (72)	1,225	IVIg utilisation study (retrospective, comparison of chronic vs. intermittent users of IVIg)	NR	NR	This was a study of IVIg utilisation. During the first year following IVIg initiation, 42.4% of IVIg initiators (*n* = 519) received ≥6 IVIg treatment courses and were therefore classified as chronic users, whereas 57.6% (*n* = 706) received 1–5 courses and were classified as intermittent users
Myasthenia gravis	Schepelmann et al., 2010 (54)	107 patients with amyotrophic lateral sclerosis (*n* = 46), myasthenia gravis (*n* = 41) or facioscapulohumeral muscular dystrophy (*n* = 20)	Patient questionnaires and diaries (non-comparative)	[For patients with all 3 diseases of the study; 12 months retrospective + 4 months prospective] 41/107 patients (38.3%) were hospitalised and 10/107 (9.3%) were treated at rehabilitation clinics due to neuromuscular diseases	NR	None
Myasthenia gravis	Sonkar et al., 2017 (30)	66	Cost analysis (prospective, non-comparative)	50 patients were admitted, and the median duration of hospitalisation was 7.5 days (range: 3–116 days)	NR	None
Myasthenia gravis	Ting et al., 2023 (61)	1,498	Analysis of healthcare resource utilisation and costs (retrospective study of insurance claims data, non-comparative)	NR	Factors that were statistically significantly associated with high healthcare resource utilisation costs included use of high-dose steroids (*p* < 0.01 vs. no use of high-dose steroids), use of chronic IVIg as second-line therapy (≥6 cycles in Year 1, *p* < 0.01 vs. acute use only or no use) or being in either the 1 or ≥4 exacerbation(s) groups (*p* < 0.02 vs. no exacerbation in Year 1)	[Mean total myasthenia gravis-related pharmacy cost over 2 years] $11,869 Study authors do not classify costs as direct or indirect
Neuro-immunological conditions (paediatric)	Nosadini et al., 2016 (73)	196	Retrospective study (chart review; includes comparison of immunoglobulin costs for patients with different neuroimmunological conditions)	NR	NR	This study provides costs for IVIg only.
NMOSD	Beekman et al., 2019 (38)	193	Cost analysis (performed within a study of patient experience and quality of life, non-comparative)	NR	NR	Cost for caregiver/support services was $4,036
NMOSD	Exuzides et al., 2021 (37)	162	Cost analysis (case-control study)	NR	NR	ED visit costs were $2,882 (SD $9,333) in patients with NMOSD and $490 ($3,097) in non-NMOSD controls. 12% of patients with NMOSD required plasmapheresis or IVIg, costing a median of $2,022 (IQR: $680–$4,584), and $29,247 ($6,515–$51,611), respectively
NMO	Holroyd et al., 2019 (40)	60 physicians	Analysis of availability and affordability of neuromyelitis optica testing and treatment (physician survey, non-comparative)	NR	NR	Average cost of an AQP4-Ab test was $287. AQP4-Ab and MOG-Ab testing was available in 68% and 38% of countries, respectively. Low-income countries had poor availability of both AQP4-Ab (2/13) and MOG-Ab (1/13) testing vs. high-income countries (15/15 and 13/15)
NMOSD	Hughes et al., 2022 (36)	117 patients and 74 informal carers	Analysis of health utilities and costs (patient survey, non-comparative)	9% of patients reported hospitalisation during the 3 months preceding questionnaire completion; mean duration of hospitalisation 12.5 days (range: 1–90 days)	Extent of disability	None
NMOSD	Knapp et al., 2022 (39)	130	Cost analysis (based on health insurance data, comparison of patients with active disease vs. inactive disease vs. controls)	NR	Active vs. inactive disease	None

*Cost data were standardised according to the cost year stated in the reference manuscript. If a cost year range was provided, the latest date was taken. If no cost year was provided, the cost year was set to the publication year. ^†^Patients with MOGAD diagnosed according to Jarius et al. (34). Study by Mahdi-Rogers et al. (55) included three different diseases (CIDP, multifocal motor neuropathy and paraproteinaemic demyelinating neuropathy), but here we have extracted data for CIDP only. The study by Schepelmann et al. (54) included three different diseases (amyotrophic lateral sclerosis, facioscapulohumeral muscular dystrophy and myasthenia gravis), but here we have extracted data for myasthenia gravis only. The publication by Allen et al. (65) is a review, retained because cost data therein appear to be original. Data inconsistencies were encountered in the publication by Winters et al. (31); for these, values from tables within the publication, rather than the main text, were selected for inclusion here. Please see Supplementary Table 2 for further details of the studies included in this table. AQP4-Ab, aquaporin-4 antibody; CASPR2, contactin-associated protein 2; CI, confidence interval; CIDP, chronic inflammatory demyelinating polyneuropathy; ED, emergency department; GABA_B_, γ-aminobutyric acid type B; HRQoL, health-related quality of life; ICD, International Classification of Diseases; ICU, intensive care unit; IgG, immunoglobulin G; IQR, interquartile range; IVIg, intravenous immunoglobulin; IVMP, intravenous methylprednisolone; LGI1, leucine-rich glioma-inactivated 1; MOG-Ab, myelin oligodendrocyte glycoprotein antibody; MOGAD, myelin oligodendrocyte glycoprotein antibody-associated disease; NMDA, N-methyl D-aspartate; NMO, neuromyelitis optica; NMOSD, neuromyelitis optica spectrum disorder; NR, not reported; SCIg, subcutaneous immunoglobulin; SD, standard deviation.

**Table 4:** Summary of publications with extractable cost estimates and cost drivers: indirect costs.

Disease	Citation	Number of patients with the disease(s) of interest	Study type	Total indirect costs	Care requirements	Care costs	Unemployment rate	Lost productivity costs	Disability rate	Disability costs	Other indirect costs	Indirect cost drivers	Additional notes
Autoimmune encephalitis	Li et al., 2020 (63)	208	Cost analysis (retrospective, non-comparative)	NR	NR	[Professional care cost, classified by the authors as a direct cost] $899 (SD $1,735)	NR	NR	NR	NR	NR	NR	None
CIDP	Mahdi-Rogers et al., 2014 (55)	43	Cost-of-illness study (patient survey, non-comparative)	$12,736 (SD $25,141)	NR	[Social services] $5,677 (SD $11,163) (UK-wide estimate $9,477,418)	NR	$12,736 (SD $25,141) (UK-wide estimate $21,254,605)	NR	NR	NR	NR	None
CIDP	McCrone et al., 2003 (52)	25	Cost–utility analysis (comparison of prednisolone vs. IVIg therapy)	NR	NR	[6-week informal care cost] $279 (SD $535) for patients treated with prednisolone and $599 ($1,105) for those treated with IVIg^†^	NR	NR	NR	NR	NR	NR	None
CIDP	Mengel et al., 2018 (53)	108	Cost-of-illness study (patient survey, non-comparative)	[3-month indirect cost] $3,305 (95% CI: $2,194–$4,606) (mean represents 17% of the mean total cost)	NR	NR	NR	[3-month costs] Premature retirement $1,635 (95% CI: $817–$2,452), unemployment $327 ($163–$654), sick leave $490 ($114–$1,026), reduced labour time $375 ($31–$791)	NR	[3-month cost] $478 (95% CI: $151–$955)	NR	NR	None
Guillain–Barré syndrome	Frenzen, 2008 (20)	[Pan-US, patients hospitalised] 5,473	Cost analysis (non-comparative)	[USA-wide] $2.380 billion (95% CI: $2.154 billion – $2,607 billion) (point estimate represents 86% of the cost of illness) [Per patient] $942,707 ($817,574–$1,067,840)	NR	NR	Projected annual number of patients in the USA not returning to work within 5 years of the onset of illness: 574 (95% CI: 512–636)	[USA-wide] $712 million (95% CI: $687 million – $738 million) (86% of the cost of illness) [Per patient] $296,379 ($265,831–$326,927)	NR	NR	[Cost for premature death, USA-wide] $1,669 billion (95% CI: $1,444 billion – $1,894 billion) (point estimate represents 86% of the cost of illness) [Cost for premature death, per patient] $6,757,397 ($5,516,566–$7,998,228)	Premature deaths	Nearly 30% of the indirect costs were due to lost productivity. Premature deaths accounted for the remainder of indirect costs and represented 60% of the total cost of Guillain–Barré syndrome. Annual number of deaths caused by Guillain–Barré syndrome in the USA: 247 (95% CI: 216–278)
Guillain–Barré syndrome	Oliveira et al., 2022 (58)	46	Cost-of-illness study (patient survey, non-comparative)	[Median total indirect cost, from symptom onset until 6 months post-discharge, total period ~6.5 months] $260 (IQR: $0–$2,165)	NR	NR	NR	[Median cost for lack of income] $0 (for the patient, accompanying person and due to premature death in each of the following 3 periods: from symptom onset to hospitalisation, during hospitalisation, and 6 months’ post-discharge)	NR	NR	NR	NR	None
Guillain–Barré syndrome	Rumalla et al., 2017 (22)	54,778	Retrospective study (review of data from the US Nationwide Inpatient Sample, comparison of patients with Guillain–Barré syndrome with vs. without hyponatraemia)	NR	NR	NR	NR	NR	Moderate to severe disability was present in 15.6% of Guillain–Barré patients with hyponatraemia, compared with 5.1% of patients with patients with Guillain–Barré syndrome without hyponatraemia (*p* < 0.0001)	NR	NR	NR	None
MOGAD^‡^ and NMOSD	Hümmert et al., 2022 (25)	212	Analysis of costs and HRQoL (patient survey, non-comparative)	$22,048 (95% CI: $16,927–$27,391) (mean value represents 23% of the mean total cost)	NR	Formal care (classified here as a direct medical cost) $5,840 (95% CI: $2,872–$10,161) Informal care (classified here as a direct non-medical cost) $26,162 ($21,041–$31,590)	NR	[Loss of salary for employed] $5,035 (95% CI: $2,913–$7,658) [Loss of salary for unemployed] $7,324 ($4,523–$10,792) [Loss of salary as an indicator for productivity loss, days of sick leave] $8,181 ($4,814–$12,127) [Loss of salary, working time reduction] $1,508 ($4,444–$2,967)	EDSS score 0–3.0, 3.5–6.0 and 6.5–8.5 in 101 (26%), 70 (50%) and 33 (11%) patients, respectively	Total cost increased with disability measured by EDSS, from $55,617 (95% CI: $45,410–$66,656) for mildly affected patients (EDSS score 0–3.0) to $206,127 ($162,035–$254,841) for severely affected patients (EDSS score 6.5–8.5). Direct medical costs, direct non-medical costs and indirect costs also increased markedly with disease severity	NR	Major drivers were loss of salary as an indicator for productivity loss, days of sick leave (37% of indirect costs) and unemployment ($7,324 [95% CI: $4,523–$10,792], 33%)	Care costs classified here as direct rather than indirect costs
Myasthenia gravis	Ignatova et al., 2022 (27)	54	Cost-of-illness study (patient survey, non-comparative)	[Median] $0	Most patients reported reliance on informal carers	[Median for professional caregiver] $0 (IQR: $0–$0) [Median for informal caregiver] $0 ($0–$0)	NR	[Median] $0 (IQR: $0–$0)	NR	NR	NR	NR	Reported social services and professional caregiver costs were very low because most patients reported reliance on informal caregivers
Myasthenia gravis	Lin et al., 2020 (21)	3,341	Cost analysis (retrospective, non-comparative)	NR	NR	NR	NR	NR	NR	NR	NR	NR	Out-of-pocket expenses increased during the study, and out-of-pocket expenses outside scope of basic medical insurance reimbursement increased from 12.6 to 18.7% of total expenses
Myasthenia gravis	Schepelmann et al., 2010 (54)	107 patients with amyotrophic lateral sclerosis (*n* = 46), myasthenia gravis (*n* = 41) or facioscapulohumeral muscular dystrophy (*n* = 20)	Patient questionnaires and diaries (non-comparative)	[From societal perspective] $5,082 (95% CI: $1,821–$12,787)	20.6% of all study participants (patients with amyotrophic lateral sclerosis, myasthenia gravis or facioscapulo-humeral muscular dystrophy) received formal care	[Informal care] $1,658 (95% CI: $219–$7,413)	NR	[Premature retirement cost for myasthenia gravis] $3,042 (95% CI: $1,530–$10,728)	NR	[Temporary disability] $383 (95% CI: $91–$1,239)	NR	NR	[For patients with all 3 diseases in the study] Average number of days per year in which patients were absent from work was 23 ± 19 (range: 2–60)
Myasthenia gravis	Sonkar et al., 2017 (30)	66	Cost analysis (prospective, non-comparative)	[Median, *n* = 66] $370 (range: $94–$16,320)	NR	NR	NR	NR	NR	NR	NR	NR	None
NMOSD	Beekman et al., 2019 (38)	193	Cost analysis (performed within a study of patient experience and quality of life, non-comparative)	NR	NR	NR	NR	[Lost income] $78,063 (largest cost, but data from only 3 patients)	NR	NR	NR	NR	None
NMOSD	Hughes et al., 2022 (36)	117 patients and 74 informal carers	Analysis of health utilities and costs (patient survey, non-comparative)	NR	NR	[Daily cost of informal care, calculated by the proxy good method or the opportunity cost method] $264 (95% central range: $33–$439) or $493 ($467–$518)	47 patients (40%) had left the workforce, including 16 (14%) due to their long-term illness and retirement	Of 13 patients (11%) in paid employment, 7 reported an average of 30 days of absence due to sickness during the 3 months before questionnaire completion	EDSS score ≤4.0, 4.5–6.5, 7.0–7.5 and 8.0–9.5 in 29 (26%), 56 (50%), 14 (13%) and 12 (11%) patients, respectively	Total cost during the 3 months preceding questionnaire completion increased with disability measured by EDSS, from $1,029 (95% central range: $698–$1,487) for the lowest EDSS category (≤4) to $59,900 ($5,288–$180,464) for the highest (EDSS 8.0–9.5). Cost for inpatient stay also increased markedly with disability, from $42 ($0–$165) to $47,513 ($0–$131,357) for the lowest and highest categories, respectively	The average 12-month cost for home adaptations was $8,867 (95% central range: $5,992–$11,739)	NR	15% of patients purchased items for home adaptations, wheelchairs and mobility scooters, public liability insurance, medication and private prescriptions during the 12 months preceding questionnaire completion
NMOSD	Knapp et al., 2022 (39)	130	Cost analysis (based on health insurance data, comparison of patients with active disease vs. inactive disease vs. controls)	NR	NR	NR	NR	NR	NR	NR	Rehabilitation $742 Aids and remedies $2,424	NR	None

*Cost data were standardised according to the cost year stated in the reference manuscript. If a cost year range was provided, the latest date was taken. If no cost year was provided, the cost year was set to the publication year. ^†^If the reporting country using Euros was not specified or if a pan-Europe study was conducted, Belgium was selected. ^‡^Patients with MOGAD diagnosed according to Jarius et al. (34). Costs shown are mean annual cost per patient unless otherwise indicated. The study by Mahdi-Rogers et al. (55) included three different diseases (CIDP, multifocal motor neuropathy and paraproteinaemic demyelinating neuropathy), but here we have extracted data for CIDP only. The study by Schepelmann et al. (54) included three different diseases (amyotrophic lateral sclerosis, facioscapulohumeral muscular dystrophy and myasthenia gravis), but here we have extracted data for myasthenia gravis only. Please see Supplementary Table 2 for further details of the studies included in this table. CI, confidence interval; CIDP, chronic inflammatory demyelinating polyneuropathy; EDSS, Expanded Disability Status Scale; HRQoL, health-related quality of life; IQR, interquartile range; IVIg, intravenous immunoglobulin; MOGAD, myelin oligodendrocyte glycoprotein antibody-associated disease; NMOSD, neuromyelitis optica spectrum disorder; SD, standard deviation; USD, US dollars.

Reported annual total cost of illness per patient, across all conditions assessed, ranged widely. The lowest annual total cost reported was a median of $3,690, based on conversion of the ~6.5 month reported cost to a full year in a Brazilian Federal District study of patients with Guillain–Barré syndrome (GBS), whereas the highest was a mean of $507,117 in a US study of GBS. Details of estimates of total annual costs are given in Table 1; for total direct costs, the corresponding low was a median of $1,981 to a high of mean $148,388 (Tables 2, 3) and for total indirect costs it was a median of $0 to a mean of $942,707 (Table 4). In most studies, direct costs appeared to comprise most (>70%) of the overall cost, although one study was an exception in this regard: a USA-wide study of GBS reported total direct medical cost as 14% of the total cost; the remaining 86% was attributable to indirect costs (20).

Reported costs for specific types of direct and indirect cost were similarly variable between studies, depending on country and disease. For example, reported costs for hospitalisation ranged from ~$0 to $93,039 (both medians) (26, 27). IVIg was an important contributor to direct medical costs in several diseases, accounting for 90% of the overall pharmacy cost in a study of CIDP and an estimated 85% of the total pharmacy cost in MG (28, 29). Several studies reported plasma exchange as less expensive or more cost-effective than IVIg (29–31), although others associated IVIg with reduced hospital stays and lower hospitalisation costs (32, 33).

In the only study of MOGAD (a cost investigation from Germany), most patients (*n* = 166) had NMOSD and 46 had MOGAD (25). The mean annualised cost of MOGAD/NMOSD was $94,688 (95% confidence interval, $81,418–$108,546), comprising direct medical costs (43%), direct non-medical costs (34%) and indirect costs (23%). Within direct medical costs, immunotherapy and inpatient hospital care were the significant drivers; those for direct non-medical costs and indirect costs were informal care and loss of salary, respectively. Need for care, number of acute attacks, unemployment, and disability measured by the Expanded Disability Status Scale (EDSS) were identified as independent predictors for cost of illness. The cost data were reported for NMOSD and MOGAD combined, as the authors found no cost differences between the two diseases stratified by disease duration and serostatus apart from in outpatient diagnostic tests (higher for MOGAD, but this was a small contributor to the overall cost of disease [<1%]). Although this study was performed prior to the publication of the 2023 MOGAD diagnostic guidelines, MOGAD was diagnosed according to international recommendations and MOG antibody positivity on a cell-based assay (34). Although studies comparing the 2023 MOGAD diagnostic criteria and the 2018 international recommendations published by Jarius et al. are sparse, one multicentre, retrospective study has found comparable sensitivity and specificity (35). One NMOSD study provided a total cost of illness with a mean annualised cost for the UK (calculated from the reported 3-month value) of $41,180 (36). In an NMOSD study from the USA, the median annual cost for IVIg was $29,247, the mean hospital cost was $34,893 and the mean cost for outpatient healthcare was $29,881 (37). Annual direct costs in NMOSD reported by other studies were $8,705 and $9,083 (mean hospital costs) (38, 39), $18,189 (mean healthcare cost) (39) and $5,248 (mean treatment cost; in this study, the term neuromyelitis optica [NMO] was used in preference to NMOSD) (40).

The authors of the MOGAD/NMOSD study compared the total cost of illness with that of MS and reported that it was higher for MOGAD/NMOSD ($94,688 vs. $65,495) despite disease severity and patient age being higher in the MS cohort (25). Our review did not include any studies focused specifically on MS.

### Cost drivers

Cost drivers were explored in numerous studies and found to include treatment (e.g., IVIg), hospitalisation, disease severity, relapses, refractory disease, active disease, disability, loss of productivity and premature death (Tables 1–4).

## Discussion

This narrative review confirms that data on cost of illness and health economic data for MOGAD are limited. We identified only one study with relevant cost data for MOGAD (25). This scenario is unsurprising given that MOGAD was only recently (October 2023) recognised as a separate entity within the ICD (8). The scarcity of economic data relating specifically to MOGAD supports our approach of examining data from analogous/proxy conditions; such methodology has been used by other groups to increase the robustness of economic modelling in rare diseases and is recognised by regulatory authorities in Europe and the USA (14–17).

Our results suggest that MOGAD and analogous conditions are associated with a range of direct and indirect costs likely to make them costly for the individual and place considerable burden on healthcare systems. As observed by Hümmert et al., the evaluation of disease-related cost is important for patients, their families and their physicians (25). It should also be a key consideration in decision-making by policy makers in the context of newly emerging treatment options for MOGAD (25). The aim of our study was to determine the overall economic impact of MOGAD. Therefore, we sought estimates of direct costs (those associated with healthcare) and indirect costs (those associated with reduced productivity) as well as the total cost. The long-term overall care requirements and effects of residual disability attributable to MOGAD are challenging to measure accurately (41–44). It is also important to note that the available economic evidence predates the new diagnostic criteria for MOGAD. Reported costs for other conditions may therefore have been affected by the inclusion of patients with MOGAD receiving an incorrect, non-MOGAD diagnosis. Unfortunately, no direct estimates of the economic impact of diagnostic insufficiencies were identified in our review. However, it is likely that an inaccurate or late diagnosis would result in delayed or inadequate treatment, slow/partial recovery between attacks and possible suboptimal management of residual disability – all of which have the potential to increase the economic burden of MOGAD. Previous studies have shown that diagnostic insufficiencies are common. It is estimated that only approximately 60% of patients receive their first consultation in less than 6 months after diagnosis, with 15% waiting over seven years; in addition, historically over half of patients have received an alternative (incorrect) diagnosis before confirmation of MOGAD (9, 45).

The extent to which results from different studies are comparable may be questioned. Costs may differ between studies according to the classification of specific elements as direct or indirect costs. Few studies provided a full and clear breakdown of either the elements included or their relative contributions to direct or indirect costs. Differences between countries in state funding can also have an effect, with costs that are borne by individual patients, their carers or their insurance companies in some countries being borne by the state healthcare system (and therefore wider society) in other countries.

In our review, the best insight into MOGAD came from a patient survey performed by Hümmert et al. in Germany (25). Economic data were reported for the whole population (*n* = 212: 46 patients with MOGAD and 166 with NMOSD), with the authors reporting little difference between the two diseases. Comparability of treatment costs for the two diseases may have been coincidental, considering the likelihood of differences in prescribing. For example, rituximab and inebilizumab are established options for patients with NMOSD but less likely to be used in MOGAD (rituximab has been shown to have reduced efficacy in MOGAD vs. NMOSD, and there are no ongoing clinical trials of inebilizumab in MOGAD) (46, 47). Additionally, maintenance therapy is usually commenced following a relapse in MOGAD; however, there is evidence that initiating maintenance therapy early, from the first attack, may substantially reduce the risk of relapse in MOGAD (48, 49), which may potentially reduce the economic burden of MOGAD.

Outpatient costs were higher with MOGAD vs. NMOSD, but Hümmert et al. attributed this to a difference in disease duration (25). Severity of disease, use of medications such as IVIg, hospitalisation costs and reduced productivity were all identified as major cost drivers (25). The authors also reported ‘need for care’, number of prior acute attacks, unemployment and disability measured using EDSS as statistically significant predictors of cost (25). An association between EDSS-measured disability and cost has also been observed in MS, consistent with the relationship between these conditions (50). Of note, Hümmert et al. observed that the cost of MOGAD/NMOSD was higher than the cost associated with MS; however, a full comparison of costs between these conditions will only be possible after MOGAD treatment guidelines and evidence are better established.

Studies in the other diseases included in our review reported findings that appeared consistent with the results of Hümmert et al. Although the NMOSD/NMO studies (36–40) reported generally lower costs than the study of MOGAD/NMOSD (25), methodological differences mean that cost variations are to be expected. Studies in CIDP and MG suggested similar drivers to those identified for MOGAD/NMOSD (30, 51–55). Studies in NMOSD, MG, CIDP and MOGAD/NMOSD reported that reduced quality of life and increased disability were associated with greater costs (36, 53, 54).

Our review suggested that a relapse can have a major impact on cost. In particular, the German study of NMOSD reported that the hospitalisation cost was >20-fold higher during the active phase (defined as the 30-day period following a relapse or period of hospitalisation for NMOSD) compared with the inactive phase (all other times) (39). Delays in diagnosis and treatment-refractory presentation may also increase the disease cost by increasing the risk of disability accrual (26, 56).

The principal limitation of this study is the sparsity of MOGAD-specific data. No study examined MOGAD alone, and only one study provided data for a mixed population of patients with NMOSD and patients with MOGAD (the majority of whom had NMOSD). Owing to differences between MOGAD and the analogous diseases included in our review, the applicability of data from other diseases to MOGAD may be questioned (variability in clinical presentation between different patients with MOGAD should also be considered here). Despite the limited quantity of available evidence and the disparate nature of studies in our review, some examples of overlap between studies in the patient populations are possible (e.g., where more than one study of one condition was conducted in a country). We acknowledge small sample sizes within studies as a further limitation. Comparisons between studies may be confounded by differences in a range of aspects such as disease severity, management approaches (for instance, novel treatments may be available for some conditions and not others) and definitions of direct vs. indirect costs. Direct costs do not differentiate between short-term treatment used to aid recovery from an attack and long-term treatments administered to prevent attacks or manage residual disability. In addition, financial structures differ considerably between countries. Most studies in our review were conducted in high-income countries, and the economic impact of MOGAD could differ in countries with lower income levels. Information from the included studies provides minimal insight into the costs of diagnosis, and there were no estimates of the economic impact of diagnostic delay or misdiagnosis. This may be related to the fact that specific testing for MOGAD became available only recently. These limitations are consistent with those typically encountered with rare diseases. In this context, Pearson et al. identified limitations in natural history and epidemiological data as key challenges in estimating the economic impact of treatment (57).

In conclusion, informal care, drugs (mainly immunotherapies) and indirect costs such as loss of income/employment are likely to be key cost contributors in the management of MOGAD. However, insight into the economic burden of MOGAD is limited by the fact that only one study has provided data from patients with this disease, and even in that study most patients had NMOSD. Further research on the economic burden of MOGAD is urgently needed.

## Data Availability

The original contributions presented in the study are included in the article/Supplementary material, further inquiries can be directed to the corresponding author.
