# Supplementary material for: A narrative review of the economic burden of myelin oligodendrocyte glycoprotein antibody-associated disease and analogous conditions
Source: Front Neurol. 2025 May 30;16:1506465. doi: 10.3389/fneur.2025.1506465 (PMC12164171; doi:10.3389/fneur.2025.1506465)
Supplement: Supplementary file 1 [file Supplementary_file_1.docx]

Supplementary information

# SUPPLEMENTARY TABLE 1 Search terms used for literature search of the PubMed database (14 July 2023).

| Thematic group | Search number | Search terms | Number of results |
| --- | --- | --- | --- |
| Cost | 1 | ("Costs and Cost Analysis"[MeSH Terms] OR "Fees and Charges"[MeSH Terms] OR "Budgets"[MeSH Terms] OR "budget*"[Title/Abstract] OR "economic*"[Title/Abstract] OR "cost"[Title/Abstract] OR "costs"[Title/Abstract] OR "costly"[Title/Abstract] OR "costing"[Title/Abstract] OR "price"[Title/Abstract] OR "prices"[Title/Abstract] OR "pricing"[Title/Abstract] OR "pharmacoeconomic*"[Title/Abstract] OR "pharmaco economic*"[Title/Abstract] OR "expenditure"[Title/Abstract] OR "expenditures"[Title/Abstract] OR "expense"[Title/Abstract] OR "expenses"[Title/Abstract] OR "financial"[Title/Abstract] OR "finance"[Title/Abstract] OR "finances"[Title/Abstract] OR "financed"[Title/Abstract] OR "value for money"[Title/Abstract] OR "monetary value*"[Title/Abstract]) | 1,099,224 |
| Economic modelling | 2 | ("models, economic"[MeSH Terms] OR "economic model*"[Title/Abstract] OR "Decision Theory"[MeSH Terms] OR "decision tree*"[Title/Abstract] OR "decision analy*"[Title/Abstract] OR "decision model*"[Title/Abstract]) OR "Economics"[MeSH Terms:noexp]) | 47,924 |
| Autoimmune conditions | 3 | ("autoimmune diseases of the nervous system"[MeSH Terms] OR ("autoimmune"[All Fields] AND "diseases"[All Fields] AND "nervous"[All Fields] AND "system"[All Fields]) OR "autoimmune diseases of the nervous system"[All Fields] OR ("autoimmune"[All Fields] AND "encephalitis"[All Fields]) OR "autoimmune encephalitis"[All Fields] OR ("oligodendrocyte myelin glycoprotein"[MeSH Terms] OR ("oligodendrocyte myelin"[All Fields] AND "glycoprotein"[All Fields]) OR "oligodendrocyte myelin glycoprotein"[All Fields] OR ("myelin"[All Fields] AND "oligodendrocyte"[All Fields] AND "glycoprotein"[All Fields]) OR "myelin oligodendrocyte glycoprotein"[All Fields] OR "myelin oligodendrocyte glycoprotein"[MeSH Terms] OR ("myelin oligodendrocyte"[All Fields] AND "glycoprotein"[All Fields]) OR "myelin oligodendrocyte glycoprotein"[All Fields] OR ("myelin"[All Fields] AND "oligodendrocyte"[All Fields] AND "glycoprotein"[All Fields]) | 94,685 |
| Exclusions | 4 | ("multiple sclerosis"[MeSH Terms] OR ("multiple"[All Fields] AND "sclerosis"[All Fields]) OR "multiple sclerosis"[All Fields]) | 84,507 |
|  | 5 | (2000/1/1:2023/6/14[pdat]) | 22,368,570 |
| Total | 6 | (((#1 OR #2) AND #3) NOT #4) AND 5 | 512 |

MeSH, medical subject heading.

### SUPPLEMENTARY TABLE 2 Further details of studies included in the review.

| **Disease(s)** | **Citation** | **Study year** | **Cost year, currency** | **Study type** | **Country / countries or region(s)** | **World Bank income level (1)** | **Number of patients with the disease(s) of interest** | **Sex (males), %** | **Age, years, median** | **Acute attack rate** |
| --- | --- | --- | --- | --- | --- | --- | --- | --- | --- | --- |
| Autoimmune encephalitis | Cai et al., 2022 (2) | 2014–2020 | CNY | Cost-efficiency analysis (retrospective, comparison of IV methylprednisolone monotherapy vs IV methylprednisolone plus IVIg combination therapy) | China | Upper middle | 78 | 61.5 | 40 (IQR, 24–58) | NR |
| Autoimmune encephalitis | Cohen et al., 2019 (3) | 2005–2015 | 2017, USD | Cost analysis (retrospective; includes comparison of antibody-positive vs. antibody-negative patients) | US | High | 63 | 46.0 | Mean, 47.2 (SE, ±2.3) | NR |
| Autoimmune encephalitis | Li et al., 2020 (4) | 2012–2018 | 2018, RMB (selected values also reported in USD) | Cost analysis (retrospective, non-comparative) | Western China | Upper middle | 208 | 49.0 | 29 (IQR, 20–44) | NR |
| Autoimmune encephalitis | Sharp et al., 2021 (5) | 2018–2019 | 2018–2019, USD | Cost analysis (relating to autoimmune encephalopathy testing panels; costs compared before and after implementing an algorithm for ordering panels) | US | High | 58 patients in 2018 and 53 patients during the first 7 months of 2019 | NR | NR | NR |
| CIDP | Allen et al., 2020 (6) | 2004–2005 and 2012–2016 | 2019, USD | Hypothetical cost analysis (reported within a review article; includes comparison of low-dose SCIg, high-dose SCIg and IVIg) | US | High | 289 | NR | NR | NR |
| CIDP | Darbà & Marsà, 2022 (7) | 2004–2018 | 2017, EUR | Cost analysis (retrospective; includes comparison of two different time periods (2004–2015 vs 2015–2016) | Spain | High | 2,805 | 64.7 | 60 (95% CI, 59–61) | NR |
| CIDP | Divino et al., 2018 (8) | 2010–2014 | 2016, USD | Cost analysis (case-control study, retrospective) | US | High | 790 | 53.7 | 52 | NR |
| CIDP | Guptill et al., 2014 (9) | 2011 | USD | Analysis of costs and healthcare resource use (retrospective, non-comparative) | US | High | 73 | 56.0 | 51 | NR |
| CIDP | Le Masson et al., 2018 (10) | 2012–2014 | 2016, EUR | Cost minimisation analysis (before and after analysis with prospective data collection; comparison of home- vs hospital-administered IVIg) | France | High | 24 | 2:1 (M:F) | Mean, 52.3 (SD, 12.2) | NR |
| CIDP | Mahdi-Rogers et al., 2014 (11) | 2008 | 2006–2007, GBP | Cost-of-illness study (patient survey, non-comparative) | UK | High | 43 | 65.1 | Mean, 65.0 (12.6) | NR |
| CIDP | McCrone et al., 2003 (12) | 1998–1999 | 2000–2001, GBR converted to EUR using Oct 2022 exchange rate | Cost–utility analysis (comparison of prednisolone vs IVIg therapy) | Belgium, Czech Republic, Greece, Italy, The Netherlands, Spain, UK | High | 25 | Prednisolone:  69  IVIg:  58 | Prednisolone:  Mean, 53.9 (SD, 17.3)  IVIg:  Mean, 52.0 (SD, 13.6 | NR |
| CIDP | Mengel et al., 2018 (13) | 2013–2014 | 2013, EUR | Cost-of-illness study (patient survey, non-comparative) | Germany | High | 108 | 77.8 | Mean, 63.1 (±12.6) | NR |
| CIDP | Perraudin et al., 2020 (14) | N/A | 2020, CHF | Cost minimalisation analysis (comparison of hospital-based IVIg vs home-based SCIg) | Switzerland | High | N/A (modelling study) | N/A | N/A | NR |
| CIDP | Piscitelli et al., 2021 (15) | N/A | NR, EUR | Cost analysis (retrospective; comparison of SCIg vs IVIg) | Italy | High | 12 | 30.8* | Mean, 56 (±15)* | NR |
| CIDP | Rajabally et al., 2019 (16) | 2014–2018 | 2018, GBP and EUR | Retrospective study (review of hospital patient records; comparison of different dosing methods for IVIg) | UK | High | 39 | 74.4 | Mean, 58.4 (SD, 15.1) | NR |
| Guillain–Barré syndrome | Frenzen, 2008 (17) | 2004 | NR, USD | Cost analysis (non-comparative) | US | High | [Pan-US, patients hospitalised] 5,473 | NR | NR | NR |
| Guillain–Barré syndrome | Maheshwari et al., 2018 (18) | 2012–2014 | 2012–2013, INR and USD | Cost minimisation analysis (includes comparison of IVIg vs plasmapheresis therapy) | India | Lower middle | 40 | 67.4 | Mean, 34.0 (SD, ± 17.1) | NR |
| Guillain–Barré syndrome | Oliveira et al., 2022 (19) | 2017–2019 | 2018, USD | Cost-of-illness study (patient survey, non-comparative) | Brazil | Upper middle | 46 | 65.5 | 42 (IQR, 28–52) | NR |
| Guillain–Barré syndrome | Rumalla et al., 2017 (20) | 2002–2011 | 2011, USD | Retrospective study (review of data from the US Nationwide Inpatient Sample, comparison of patients with Guillain–Barré syndrome with vs without hyponatraemia) | US | High | 54,778 | No hyponatremia:  55.9  Hyponatremia:  52.6 | No hyponatremia:  Mean, 52.6 (SD, 17.8)  Hyponatremia:  Mean, 59.5 (SD, 16.3) | NR |
| Guillain–Barré syndrome | Tsai et al., 2007 (21) | 1999–2004 | 2005, NTD | Cost-effectiveness study (retrospective; includes comparison of IVIg vs plasma exchange therapy) | Taiwan | High | 24 | PLEX:  70  IVIg:  86  Control:  86 | PLEX:  Mean, 61.0 ± 17.4  IVIg:  Mean, 45.0 ± 25.0  Control:  Mean, 49.1 ± 23.0 | NR |
| Guillain–Barré syndrome | van Leeuwen et al., 2016 (22) | 2009–2010 | EUR | Analysis of resource use and costs (non-comparative) | The Netherlands | High | 87 | 56.0 | 49 (IQR, 30–64) | NR |
| Guillain–Barré syndrome | Winters et al., 2011 (23) | 2010–2011 | 2011, USD | Cost minimisation analysis (comparison of IVIg vs plasma exchange) | US | High | Not applicable (modelling study) | NR | NR | NR |
| MOGAD and NMOSD^†^ | Hümmert et al., 2022 (24) | 2017–2019 | 2018, EUR | Analysis of costs and HRQoL (patient survey, non-comparative) | Germany | High | 212 | 20 | 50 (range, 19–83) | NR |
| Myasthenia gravis | Fan et al., 2020 (25) | Not reported | CNY | HRQoL study (cross-sectional, non-comparative) | China | Upper middle | 69 | 62.3 | Mean, 54.7 (±13.7) | NR |
| Myasthenia gravis | Guptill et al., 2011 (26) | 2008–2010 | USD | Cost analysis (includes comparison of costs for IVIg vs plasma exchange therapy) | US | High | 1,288 | 41 | Mean, 59.8 | NR |
| Myasthenia gravis | Guptill et al., 2012 (27) | 2009 | USD | Cost analysis (case-control study) | US | High | 113 | 35 | Mean, 53 | NR |
| Myasthenia gravis | Harris et al., 2020 (28) | 2013–2019 | NR | Healthcare resource utilisation study (retrospective, non-comparative) | US | High | 782 | Ever-refractory gMG:  27  Non-refractory gMG:  43 | Every-refractory gMG:  Mean, 51.6 (SD, 14.3)  Non-refractory gMG:  Mean, 59.2 (SD, 13.9) | 6-month probability of an exacerbation decreased over time, from ~60% at baseline to ~20–40% at 4 years |
| Myasthenia gravis | Ignatova et al., 2022 (29) | 2020 | 2020, EUR | Cost-of-illness study (patient survey, non-comparative) | Bulgaria | High | 54 | 19 | Mean, 45 (SD, 13) | NR |
| Myasthenia gravis | Lin et al., 2020 (30) | 2013–2015 | [Costs expressed as percentages of total expenses; actual values not provided] | Cost analysis (retrospective, non-comparative) | China | Upper middle | 3,341 | 41.6 | 50–59, 22.7%  60–69, 20.6%  70–79, 16.9% | NR |
| Myasthenia gravis | Mandawat et al., 2010 (31) | 2000–2005 | Year not reported, USD | Economic outcomes study (retrospective, comparison of patients with myasthenia gravis with vs without crisis) | US | High | 1,606 (908 patients with myasthenia gravis and 698 patients with myasthenia gravis crisis) | Myasthenia Gravis:  PLEX, 34.3  IVIG, 37.4  Myasthenia Gravis crisis:  PLEX, 45.4  IVIG, 29.6 | Myasthenia Gravis:  PLEX, mean, 53.2 (SD, 18.4)  IVIg, mean, 50.7 (SD, 23.7)  Myasthenia Gravis crisis:  PLEX, mean, 58.9 (SD, 18.5)  IVIg, mean, 56.3 (SD, 22.0) | NR |
| Myasthenia gravis | Qi et al., 2022 (32) | 2014–2019 | 2018, USD | IVIg utilisation study (retrospective, comparison of chronic versus intermittent users of IVIg) | US | High | 1,225 | 43.1 | Mean, 58.9 (SD, 14.8) | NR |
| Myasthenia gravis | Schepelmann et al., 2010 (33) | 2005 | 2009, EUR | Patient questionnaires and diaries (non-comparative) | Germany | High | myasthenia gravis (*n* = 41) | 43.9 | 51–70, 29.3%  >70, 36.6% | NR |
| Myasthenia gravis | Sonkar et al., 2017 (34) | 2014–2016 | 2016, INR and USD | Cost analysis (prospective, non-comparative) | India | Lower middle | 66 | 59.0 | 42 (range, 6–75) | NR |
| Myasthenia gravis | Ting et al., 2023 (35) | 2008–2019 | 2019, USD | Analysis of healthcare resource utilisation and costs (retrospective study of insurance claims data, non-comparative) | US | High | 1,498 | 46.9 | 59.0 (IQR, 48.0–71.0) | During the 1-year baseline period, there was a mean of 0.95 exacerbations per patient (SD 3.18). Over the 2-year follow-up period, 49% of the total cohort had ≥1 myasthenia gravis exacerbation and, among these patients, the mean number of exacerbations per patient was 5.10 (10.55). The mean number of claims per patient among the total population was 2.49 (7.80) |
| Neuroimmunological conditions (paediatric) | Nosadini et al., 2016 (36) | 2000–2014 | 2015, USD | Retrospective study (chart review; includes comparison of immunoglobulin costs for patients with different neuroimmunological conditions) | Australia | High | 196 | 49.0 | 5.1 (range, 0.3–15.8) | NR |
| NMOSD | Beekman et al., 2019 (37) | Not reported | Year NR, USD | Cost analysis (performed within a study of patient experience and quality of life, non-comparative) | US | High | 193 | 11.4 | Mean, 49.2 ± 12.8 (range, 19–76) | NR |
| NMOSD | Exuzides et al., 2021 (38) | 2014–2018 | 2019, USD | Cost analysis (case-control study) | US | High | 162 | 26.5 | 45 (Q1–Q3, 33–58) | NR |
| NMO | Holroyd et al., 2019 (39) | 2018 | 2011, USD | Analysis of availability and affordability of neuromyelitis optica testing and treatment (physician survey, non-comparative) | Global | High, upper middle, lower middle and low | 60 physicians | N/A | N/A | NR |
| NMOSD | Hughes et al., 2022 (40) | 2016–2018 | 2016–2017, GBP | Analysis of health utilities and costs (patient survey, non-comparative) | UK | High | 117 patients and 74 informal carers | 22 | Mean, 53 (SD, 15) | Mean of 3 relapses (range 0–10) after their first attack since diagnosis; mean duration since first attack of 12 years (SD 8 years) |
| NMOSD | Knapp et al., 2022 (41) | 2013–2019 | Year NR, EUR | Cost analysis (based on health insurance data, comparison of patients with active disease vs inactive disease vs controls) | Germany | High | 130 | 42 | 46.5 (range, 3–89) | NR |

*Sex and age data include 13 patients that received IVIG treatment and were selected to switch to SCIG therapy. One patient returned to IVIG therapy after symptoms worsened. ^†^Patients with MOGAD diagnosed according to Jarius et al. 2018 (42).
The study by Mahdi-Rogers et al. (11) included three different diseases (CIDP, multifocal motor neuropathy and paraproteinaemic demyelinating neuropathy), but here we have extracted data for chronic inflammatory demyelinating polyneuropathy only. The study by Schepelmann et al. (33) included three different diseases (amyotrophic lateral sclerosis, facioscapulohumeral muscular dystrophy and myasthenia gravis), but here we have extracted data for myasthenia gravis only. The publication by Allen et al. (6) is a review, retained because cost data therein appear to be original (study dates have been taken from the PATH and ICE studies, and the number of patients is the sum of participants from these two studies). CHF, Swiss francs; CIDP, chronic inflammatory demyelinating polyneuropathy; CNY, Chinese yuan; EUR, Euros; GBP, British pounds; HRQoL, health-related quality of life; INR, Indian rupees; IV, intravenous; IVIg, intravenous immunoglobulin; MOGAD, myelin oligodendrocyte glycoprotein antibody-associated disease; NMO, neuromyelitis optica; NMOSD, neuromyelitis optica spectrum disorder; NR, not reported; NTD, new Taiwan dollars; PLEX, plasma exchange; RMB, renminbi; SCIg, subcutaneous immunoglobulin; SD, standard deviation; USD, US dollars.

**References**

1. World Bank. (2024). World Bank country classifications by income level for 2024–2025. <https://blogs.worldbank.org/en/opendata/world-bank-country-classifications-by-income-level-for-2024-2025> [Accessed October 4, 2024].

2. Cai MT, Lai QL, Zheng Y, Fang GL, Shen CH, Xu YF, et al. First-line immunotherapy of neuronal surface antibody-mediated autoimmune encephalitis: assessment of therapeutic effectiveness and cost-efficiency. *Mult Scler Relat Disord.* (2022) 66:104071. doi: 10.1016/j.msard.2022.104071

3. Cohen J, Sotoca J, Gandhi S, Yeshokumar AK, Gordon-Lipkin E, Geocadin RG, et al. Autoimmune encephalitis: a costly condition. *Neurology.* (2019) 92:e964–72. doi: 10.1212/WNL.0000000000006990

4. Li A, Gong X, Guo K, Lin J, Zhou D, Hong Z. Direct economic burden of patients with autoimmune encephalitis in western China. *Neurol Neuroimmunol Neuroinflamm.* (2020) 7:e891. doi: 10.1212/NXI.0000000000000891

5. Sharp CN, Fletcher A, Muluhngwi P, Snyder J, Linder MW, Jortani SA. A shared diagnostic stewardship approach toward improving autoimmune encephalopathy send-out testing utilization. *J Appl Lab Med.* (2021) 6:387–96. doi: 10.1093/jalm/jfaa123

6. Allen JA, Gelinas DF, Freimer M, Runken MC, Wolfe GI. Immunoglobulin administration for the treatment of CIDP: IVIG or SCIG? *J Neurol Sci.* (2020) 408:116497. doi: 10.1016/j.jns.2019.116497

7. Darbà J, Marsà A. Chronic inflammatory demyelinating polyneuropathy in Spain: a retrospective analysis of hospital incidence and medical costs. *Expert Rev Pharmacoecon Outcomes Res.* (2022) 22:665–70. doi: 10.1080/14737167.2022.2000862

8. Divino V, Mallick R, DeKoven M, Krishnarajah G. The economic burden of CIDP in the United States: a case-control study. *PLoS One.* (2018) 13:e0206205. doi: 10.1371/journal.pone.0206205

9. Guptill JT, Bromberg MB, Zhu L, Sharma BK, Thompson AR, Krueger A, et al. Patient demographics and health plan paid costs in chronic inflammatory demyelinating polyneuropathy. *Muscle Nerve.* (2014) 50:47–51. doi: 10.1002/mus.24109

10. Le Masson G, Sole G, Desnuelle C, Delmont E, Gauthier-Darnis M, Puget S, et al. Home versus hospital immunoglobulin treatment for autoimmune neuropathies: a cost minimization analysis. *Brain Behav.* (2018) 8:e00923. doi: 10.1002/brb3.923

11. Mahdi-Rogers M, McCrone P, Hughes RA. Economic costs and quality of life in chronic inflammatory neuropathies in southeast England. *Eur J Neurol.* (2014) 21:34–9. doi: 10.1111/ene.12245

12. McCrone P, Chisholm D, Knapp M, Hughes R, Comi G, Dalakas MC, et al. Cost-utility analysis of intravenous immunoglobulin and prednisolone for chronic inflammatory demyelinating polyradiculoneuropathy. *Eur J Neurol.* (2003) 10:687–94. doi: 10.1046/j.1351-5101.2003.00701.x

13. Mengel D, Fraune L, Sommer N, Stettner M, Reese JP, Dams J, et al. Costs of illness in chronic inflammatory demyelinating polyneuropathy in Germany. *Muscle Nerve.* (2018) 58:681–7. doi: 10.1002/mus.26315

14. Perraudin C, Bourdin A, Vicino A, Kuntzer T, Bugnon O, Berger J. Home-based subcutaneous immunoglobulin for chronic inflammatory demyelinating polyneuropathy patients: a Swiss cost-minimization analysis. *PLoS One.* (2020) 15:e0242630. doi: 10.1371/journal.pone.0242630

15. Piscitelli E, Massa M, De Martino BM, Serio CS, Guglielmi G, Colacicco G, et al. Economic evaluation of subcutaneous versus intravenous immunoglobulin therapy in chronic inflammatory demyelinating polyneuropathy: a real-life study. *Eur J Hosp Pharm.* (2021) 28:e115–9. doi: 10.1136/ejhpharm-2020-002430

16. Rajabally YA, Afzal S. Clinical and economic comparison of an individualised immunoglobulin protocol vs. standard dosing for chronic inflammatory demyelinating polyneuropathy. *J Neurol.* (2019) 266:461–7. doi: 10.1007/s00415-018-9157-4

17. Frenzen PD. Economic cost of Guillain–Barré syndrome in the United States. *Neurology.* (2008) 71:21–7. doi: 10.1212/01.wnl.0000316393.54258.d1

18. Maheshwari A, Sharma RR, Prinja S, Hans R, Modi M, Sharma N, et al. Cost-minimization analysis in the Indian subcontinent for treating Guillain Barre Syndrome patients with therapeutic plasma exchange as compared to intravenous immunoglobulin. *J Clin Apher.* (2018) 33:631–7. doi: 10.1002/jca.21646

19. Oliveira AFM, Gallo LG, Bastos MM, Abrahão AA, Garcia KKS, de Carvalho JKS, et al. Costs of Guillain–Barré syndrome in the Brazilian Federal District: the patients' perspective. *Trans R Soc Trop Med Hyg.* (2022) 116:310–21. doi: 10.1093/trstmh/trab118

20. Rumalla K, Reddy AY, Letchuman V, Mittal MK. Hyponatremia in Guillain–Barré syndrome. *J Clin Neuromuscul Dis.* (2017) 18:207–17. doi: 10.1097/cnd.0000000000000157

21. Tsai CP, Wang KC, Liu CY, Sheng WY, Lee TC. Pharmacoeconomics of therapy for Guillain–Barré syndrome: plasma exchange and intravenous immunoglobulin. *J Clin Neurosci.* (2007) 14:625–9. doi: 10.1016/j.jocn.2006.03.020

22. van Leeuwen N, Lingsma HF, Vanrolleghem AM, Sturkenboom MC, van Doorn PA, Steyerberg EW, et al. Hospital admissions, transfers and costs of Guillain–Barré syndrome. *PLoS One.* (2016) 11:e0143837. doi: 10.1371/journal.pone.0143837

23. Winters JL, Brown D, Hazard E, Chainani A, Andrzejewski C, Jr. Cost-minimization analysis of the direct costs of TPE and IVIg in the treatment of Guillain–Barré syndrome. *BMC Health Serv Res.* (2011) 11:101. doi: 10.1186/1472-6963-11-101

24. Hümmert MW, Schöppe LM, Bellmann-Strobl J, Siebert N, Paul F, Duchow A, et al. Costs and health-related quality of life in patients with NMO spectrum disorders and MOG-antibody-associated disease: CHANCE(NMO) Study. *Neurology.* (2022) 98:e1184–96. doi: 10.1212/wnl.0000000000200052

25. Fan X, Xing C, Yang L, Wang J, Feng L. Fatigue, self-efficacy and psychiatric symptoms influence the quality of life in patients with myasthenia gravis in Tianjin, China. *J Clin Neurosci.* (2020) 79:84–9. doi: 10.1016/j.jocn.2020.06.023

26. Guptill JT, Marano A, Krueger A, Sanders DB. Cost analysis of myasthenia gravis from a large U.S. insurance database. *Muscle Nerve.* (2011) 44:907–11. doi: 10.1002/mus.22212

27. Guptill JT, Sharma BK, Marano A, Soucy A, Krueger A, Sanders DB. Estimated cost of treating myasthenia gravis in an insured U.S. population. *Muscle Nerve.* (2012) 45:363–6. doi: 10.1002/mus.22327

28. Harris L, Allman PH, Sheffield R, Cutter G. Longitudinal analysis of disease burden in refractory and nonrefractory generalized myasthenia gravis in the United States. *J Clin Neuromuscul Dis.* (2020) 22:11–21. doi: 10.1097/cnd.0000000000000301

29. Ignatova V, Kostadinov K, Vassileva E, Muradyan N, Stefanov G, Iskrov G, et al. Socio-economic burden of myasthenia gravis: a cost-of-illness study in Bulgaria. *Front Public Health.* (2022) 10:822909. doi: 10.3389/fpubh.2022.822909

30. Lin T-Y, Zhang X-Y, Fang P-Q, Min R. Out-of-pocket expenses for myasthenia gravis patients in China: a study on patients insured by basic medical insurance in China, 2013–2015. *Orphanet J Rare Dis.* (2020) 15:13. doi: 10.1186/s13023-019-1289-9

31. Mandawat A, Kaminski HJ, Cutter G, Katirji B, Alshekhlee A. Comparative analysis of therapeutic options used for myasthenia gravis. *Ann Neurol.* (2010) 68:797–805. doi: 10.1002/ana.22139

32. Qi CZ, Hughes T, Gelinas D, Li Y, Goyal A, Brauer E, et al. Real-world utilization patterns of intravenous immunoglobulin in adults with generalized myasthenia gravis in the United States. *J Neurol Sci.* (2022) 443:120480. doi: 10.1016/j.jns.2022.120480

33. Schepelmann K, Winter Y, Spottke AE, Claus D, Grothe C, Schröder R, et al. Socioeconomic burden of amyotrophic lateral sclerosis, myasthenia gravis and facioscapulohumeral muscular dystrophy. *J Neurol.* (2010) 257:15–23. doi: 10.1007/s00415-009-5256-6

34. Sonkar KK, Bhoi SK, Dubey D, Kalita J, Misra UK. Direct and indirect cost of myasthenia gravis: a prospective study from a tertiary care teaching hospital in India. *J Clin Neurosci.* (2017) 38:114–7. doi: 10.1016/j.jocn.2016.11.003

35. Ting A, Story T, Lecomte C, Estrin A, Syed S, Lee E. A real-world analysis of factors associated with high healthcare resource utilization and costs in patients with myasthenia gravis receiving second-line treatment. *J Neurol Sci.* (2023) 445:120531. doi: 10.1016/j.jns.2022.120531

36. Nosadini M, Mohammad SS, Suppiej A, Sartori S, Dale RC. Intravenous immunoglobulin in paediatric neurology: safety, adherence to guidelines, and long-term outcome. *Dev Med Child Neurol.* (2016) 58:1180–92. doi: 10.1111/dmcn.13159

37. Beekman J, Keisler A, Pedraza O, Haramura M, Gianella-Borradori A, Katz E, et al. Neuromyelitis optica spectrum disorder: patient experience and quality of life. *Neurol Neuroimmunol Neuroinflamm.* (2019) 6:e580. doi: 10.1212/nxi.0000000000000580

38. Exuzides A, Sheinson D, Sidiropoulos P, Gholizadeh S, Magrini F, Surinach A, et al. The costs of care from a US claims database in patients with neuromyelitis optica spectrum disorder. *J Neurol Sci.* (2021) 427:117553. doi: 10.1016/j.jns.2021.117553

39. Holroyd K, Vogel A, Lynch K, Gazdag B, Voghel M, Alakel N, et al. Neuromyelitis optica testing and treatment: availability and affordability in 60 countries. *Mult Scler Relat Disord.* (2019) 33:44–50. doi: 10.1016/j.msard.2019.05.013

40. Hughes DA, Bourke S, Jones A, Bhatt R, Huda S, Mutch K, et al. Health utilities and costs for neuromyelitis optica spectrum disorder. *Orphanet J Rare Dis.* (2022) 17:159. doi: 10.1186/s13023-022-02310-z

41. Knapp RK, Hardtstock F, Wilke T, Maywald U, Deiters B, Schneider S, et al. Evaluating the economic burden of relapses in neuromyelitis optica spectrum disorder: a real-world analysis using German claims data. *Neurol Ther.* (2022) 11:247–63. doi: 10.1007/s40120-021-00311-x

42. Jarius S, Paul F, Aktas O, Asgari N, Dale RC, de Seze J, et al. MOG encephalomyelitis: international recommendations on diagnosis and antibody testing. *J Neuroinflammation.* (2018) 15:134. doi: 10.1186/s12974-018-1144-2
